# Comparison of Chemical Compositions and Antioxidant Activity of Essential Oils from Litsea Cubeba, Cinnamon, Anise, and Eucalyptus

**DOI:** 10.3390/molecules28135051

**Published:** 2023-06-28

**Authors:** Shutian Liu, Chen Zhao, Yuwei Cao, Yan Li, Zhuo Zhang, Dechao Nie, Weixuan Tang, Yanling Li

**Affiliations:** Animal Science and Technology College, Beijing University of Agriculture, No. 7 Beinong Road, Changping, Beijing 102206, China; liushutian622@163.com (S.L.); zhaochenunique@163.com (C.Z.); cyw2222021@126.com (Y.C.); liyan06122022@163.com (Y.L.); zzhuo0126@163.com (Z.Z.); niedechao0403@163.com (D.N.); winsometang@163.com (W.T.)

**Keywords:** litsea cubeba oil, cinnamon oil, eucalyptus oil, anise oil, antioxidant activity

## Abstract

The purpose of this study was to compare the antioxidant activity of litsea cubeba oil (LCO), cinnamon oil (CO), anise oil (AO), and eucalyptus oil (EUC) in vitro. The chemical compositions of the essential oils (EOs) were analyzed using gas chromatography-mass spectrometry (GC-MS). The antioxidant activity of the four EOs was evaluated through scavenging DPPH free radicals, chelating Fe^2+^, scavenging hydroxyl free radicals, and inhibiting yolk lipid peroxidation. The revised compositional analysis showed that the major constituents of LCO, CO, AO, and EUC were citral (geranial plus neral, 63.55%), (E)-cinnamaldehyde (84.86%), (E)-anethole (78.51%), and 1,8-cineole (81.79%), respectively. The four EOs all had certain antioxidant activity. The ability to scavenge DPPH radical was ranked in the order of LCO > CO > AO > EUC. The hydroxyl radical scavenging ability was ranked in the order of EUC > CO > LCO > AO. The chelating Fe^2+^ capacity was ranked in the order of EUC > AO > CO > LCO. The yolk lipid peroxidation inhibition ability was ranked in the order of CO > AO > EUC > LCO. In different antioxidant activity assays, the antioxidant activity of the EOs was different. It was speculated that the total antioxidant activity of an EO may be the result of the joint action of different antioxidant capacities.

## 1. Introduction

In livestock production, intensive farming patterns and harmful environmental factors (e.g., bacteria) can lead to oxidative stress in animals. The overproduction of reactive oxygen species and an imbalance of antioxidant systems can cause oxidative stress [1]. Oxidative stress can destroy a variety of cellular components via DNA hydroxylation, protein denaturation, lipid peroxidation, and membrane rupture, which results in cell apoptosis and other cell death patterns [2,3]. Oxidative stress causes oxidative damage to the digestive tract, which, in turn, induces an inflammatory response in the organism. This ultimately leads to stunted growth, reduced immunity, and even death in the animal, which can seriously affect economic efficiency [4]. Eliminating and slowing down oxidative stress is a key step in protecting animals from injury. Some synthetic antioxidants, such as butylhydroxytoluene (BHT) and butylhydroxyanisole (BHA), may have carcinogenic potential and pose a risk to animal health and product quality. Therefore, it is important to find natural green antioxidants to reduce oxidative stress in animals.

Essential oils (EOs) are aromatic and volatile mixtures composed of dozens to hundreds of compounds, among which monoterpenes, sesquiterpenes, and their oxygen-containing derivatives are the main components [2,3,5]. The antioxidative and antibacterial activities of EOs are derived from these components. The toxicity and side effects of EOs are low, meaning they can be widely used in animal husbandry [6]. As a natural antioxidant, EOs have been used in livestock and poultry production. Previous studies have reported that EOs reduce fat oxidation and improve the shelf-life of meat quality [7]. EOs can alleviate oxidative damage and inhibit the activation of NF-κB signaling pathways, thereby alleviating the damage of immune functions caused by oxidative stress [8,9]. Other studies have reported that EO supplementation increased the activity of antioxidant enzymes in the body and improved the growth performance and immune functions of animals [10]. Therefore, the antioxidant activity of EOs has important biological significance [11]. Some studies have indicated that *Litsea cubeba* (Lour.) Pers. oil (LCO), *Cinnamomum cassia* (L.) D. Don oil (CO), *Pimpinella anisum* L. oil (AO), and *Eucalyptus* spp. oil (EUC) have antibacterial, anti-inflammatory, and antioxidant activities, and these four EOs are widely used in the pharmaceutical and food industries in China [12,13,14,15]. Thus, the purpose of this study was to evaluate the chemical compositions of LCO, CO, AO, and EUC and compare their antioxidant activity via a DPPH radical scavenging assay, Fe^2+^ chelating assay, hydroxyl radical scavenging assay, and yolk lipid peroxidation assay. This study provides a theoretical basis for the application of EOs as a natural antioxidant in animal husbandry.

## 2. Results

### 2.1. Chemical Compositions of Essential Oils

The chemical compositions of the four EOs are shown in Table 1. 30, 20, 20, and 13 peaks were detected in LCO, CO, AO, and EUC, respectively. LCO was dominated by geranial (35.58%), neral (27.97%), and limonene (12.39%); geranial and neral together accounted for 63.55% citral. An unresolved oxygenated monoterpene peak accounted for 2.62%. The major constituents of CO were (E)-cinnamaldehyde (84.86%), tentative coumarin (6.43%), tentative 2-methoxycinnamaldehyde (1.76%), and cinnamyl acetate (1.46%). AO contained (E)-anethole (78.51%), estragole (6.15%), tentative foeniculin (3.47%), linalool (2.21%), and limonene (2.20%). EUC contained 1,8-cineole (81.79%), tentative 4-carene (5.35%), β-myrcene (4.58%), sabinene (2.19%), and α-phellandrene (1.97%). Identification confidence (ID) and unresolved assignments are indicated in Table 1.

### 2.2. The Antioxidant Activity of Essential Oils

#### 2.2.1. Effect of EOs on DPPH Radical Scavenging Rates

**Figure 1:**
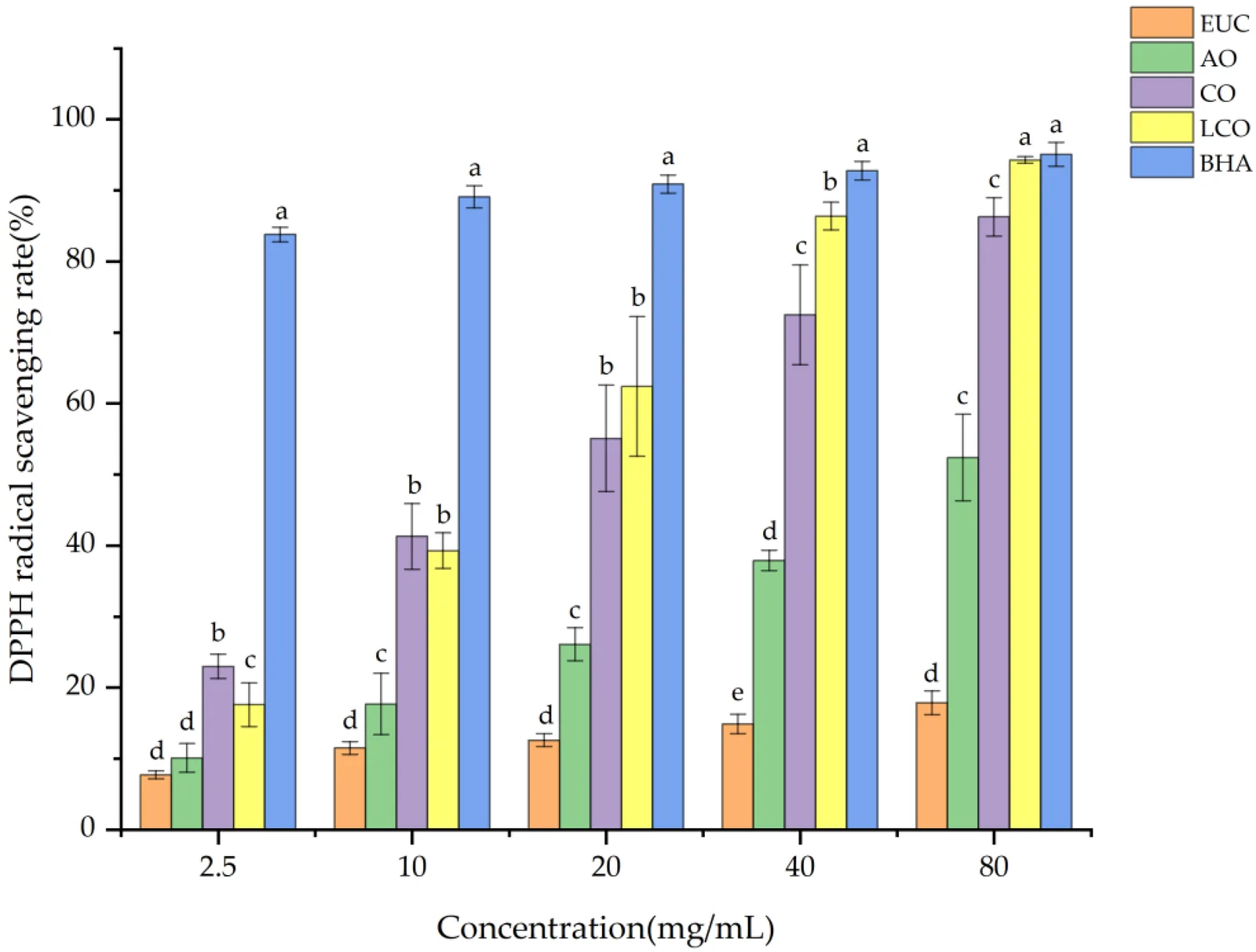
Effects of different concentrations of EOs on DPPH radical scavenging rates. EOs (E) and their different concentrations (C) had significant effects on DPPH radical scavenging rates (*p_E_* < 0.01, *p_C_* < 0.01), and their interaction (E × C) was significant (*p_E_* × *p_C_* < 0.01). Different letters show differences (*p* < 0.05) among EOs. LCO: litsea cubeba oil, CO: cinnamon oil, AO: anise oil, EUC: eucalyptus oil; BHA: butylated hydroxyanisole.

Figure 1 shows the effects of the EO type and the EO concentration on the scavenging rates of DPPH radicals. EO type (E) and EO concentration (C) had significant effects on DPPH radical scavenging rates (*p_E_* < 0.01, *p_C_* < 0.01), and the interactions between EO type and concentration (E × C) were significant (*p_E_* × *p_C_* < 0.01). The DPPH radical scavenging rate quadratically increased (*p*_Q_ < 0.01) with an increase in the concentration of LCO, CO, AO, and EUC. In comparing rates among EOs, the DPPH radical scavenging rate differed (*p* < 0.05) at a concentration of 2.5 mg/mL (CO > LCO > AO, EUC), at a concentration of 10 and 20 mg/mL (CO, LCO > AO > EUC), at a concentration of 40 mg/mL (LCO > CO > AO > EUC), and at a concentration of 80 mg/mL (LCO > CO > AO > EUC). The BHA had consistently greater *(p* < 0.05) hydroxyl radical scavenging rates compared to all EOs, except there was no significant difference (*p* > 0.05) in DPPH radical scavenging between LCO and BHA at the concentration of 80 mg/mL.

#### 2.2.2. Effect of EOs on Fe^2+^ Chelating Ability

**Figure 2:**
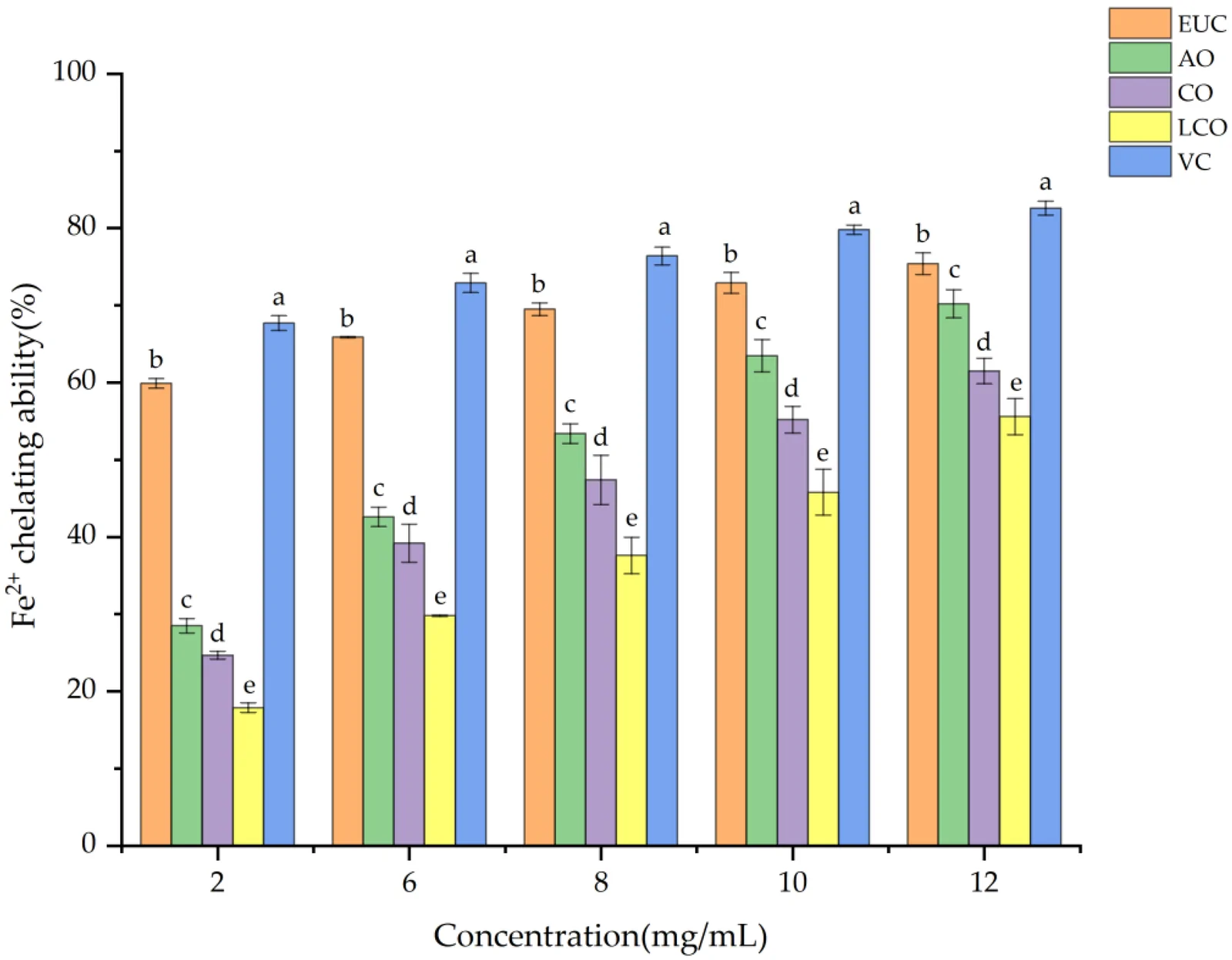
Effects of different concentrations of EOs on Fe^2+^ chelating ability. EOs (E) and their different concentrations (C) had significant effects on Fe^2+^ chelating capacity (*p_E_* < 0.01, *p_C_* < 0.01), and their interaction (E × C) was significant (*p_E_* × *p_C_* < 0.01). Different letters show differences (*p* < 0.05) among EOs. LCO: litsea cubeba oil, CO: cinnamon oil, AO: anise oil, EUC: eucalyptus oil; VC: Vitamin C.

Figure 2 shows the effects of the EO type and the EO concentration on Fe^2+^ chelating capacity. EO type (E) and EO concentration (C) had significant effects on the Fe^2+^ chelating ability (*p_E_* < 0.01, *p_C_* < 0.01), and the interactions between EO type and concentration (E × C) were significant (*p_E_* × *p_C_* < 0.01). The Fe^2+^ chelating capacity quadratically increased (*p*_Q_ < 0.01) with an increase in the concentration of LCO, and linearly increased (*p_L_* < 0.01) with an increase in the concentration of CO, AO, and EUC. In comparing capacity among EOs, the Fe^2+^ chelating capacity differed (*p* < 0.05) at five different EO concentration levels (2, 6, 8, 10, and 12 mg/mL) (EUC > AO > CO > LCO). The VC had a consistently greater (*p* < 0.05) Fe^2+^ chelating capacity compared to all EOs at all of the concentrations. At the concentration of 12 mg/mL, the chelation rate of VC to Fe^2+^ was 82.59%, and the chelation rates of AO and EUC to Fe^2+^ were 70.15% and 75.39%, respectively, reaching 84.94% and 91.28% of VC.

#### 2.2.3. Effect of EOs on Hydroxyl Radical Scavenging Rates

**Figure 3:**
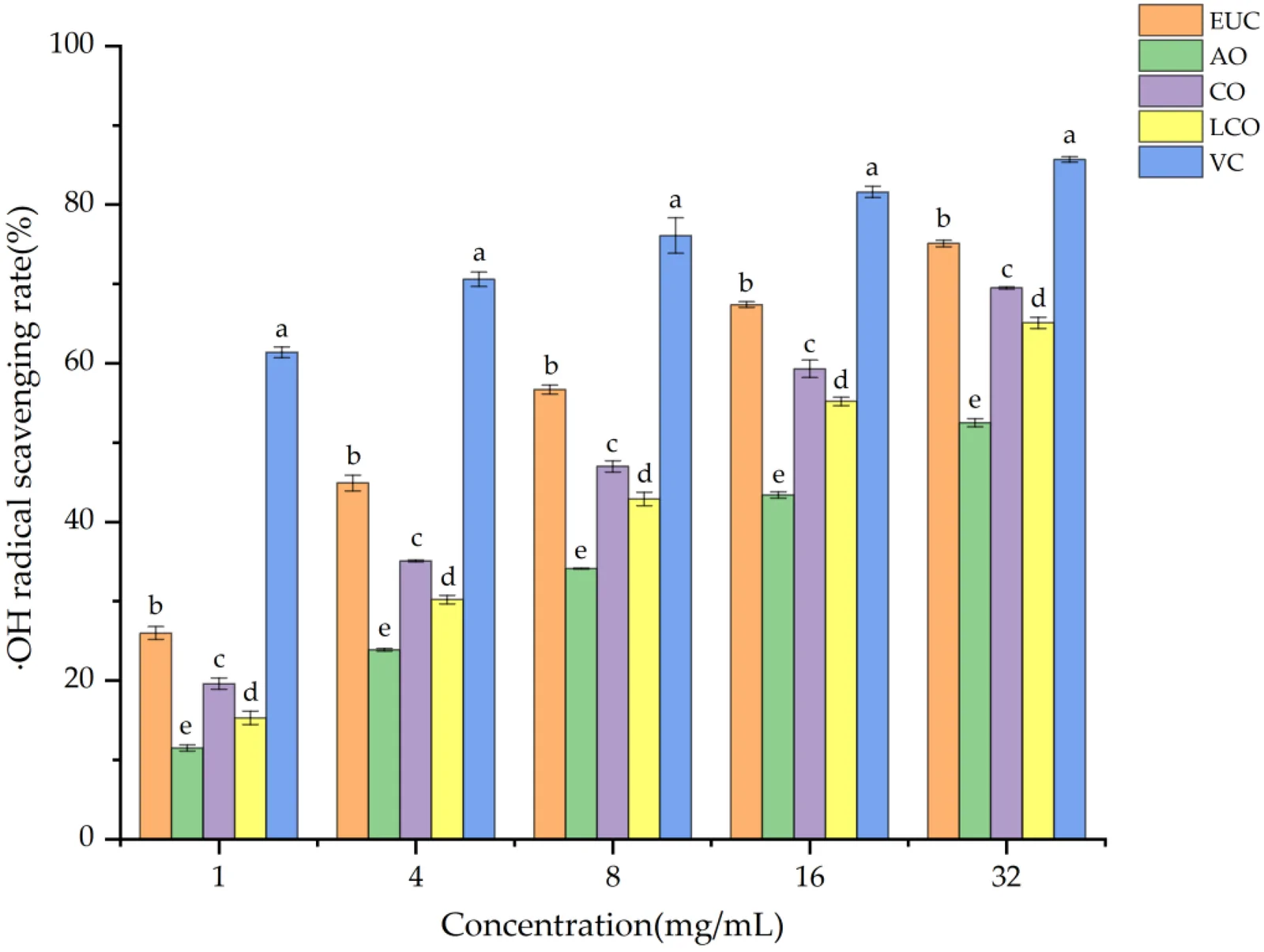
Effects of different concentrations of EOs on ·OH radical scavenging rates. EOs (E) and their different concentrations (C) had significant effects on ·OH radical scavenging rates (*p_E_* < 0.01, *p_C_* < 0.01), and their interaction (E × C) was significant (*p_E_* × *p_C_* < 0.01). Different letters show differences (*p* < 0.05) among EOs. LCO: litsea cubeba oil, CO: cinnamon oil, AO: anise oil, EUC: eucalyptus oil; VC: Vitamin C.

The effects of the EO type and the EO concentration on the scavenging rates of hydroxyl radicals are shown in Figure 3. EO type (E) and EO concentration (C) had significant effects on the hydroxyl radical scavenging rate (*p_E_* < 0.01, *p_C_* < 0.01), and the interactions between EO type and concentration (E × C) were significant (*p_E_ × p_C_* < 0.01). The hydroxyl radical scavenging rate quadratically increased (*p*_Q_ < 0.01) with an increase in the concentration of LCO, CO, AO, and EUC. In comparing rates among EOs, the hydroxyl radical scavenging rate differed (*p* < 0.05) at five different EO concentration levels (1, 4, 8, 16, and 32 mg/mL) (EUC > CO > LCO > AO). The VC had a consistently greater (*p* < 0.05) hydroxyl radical scavenging rate compared to all EOs at all of the concentrations. At the concentration of 32 mg/mL, the hydroxyl radical scavenging rate of VC was 85.66%, and the hydroxyl radical scavenging rate of EUC was 75.05%, which reached more than 80% of the level of VC.

#### 2.2.4. Effects of EOs on the Inhibition of Yolk Lipid Peroxidation

**Figure 4:**
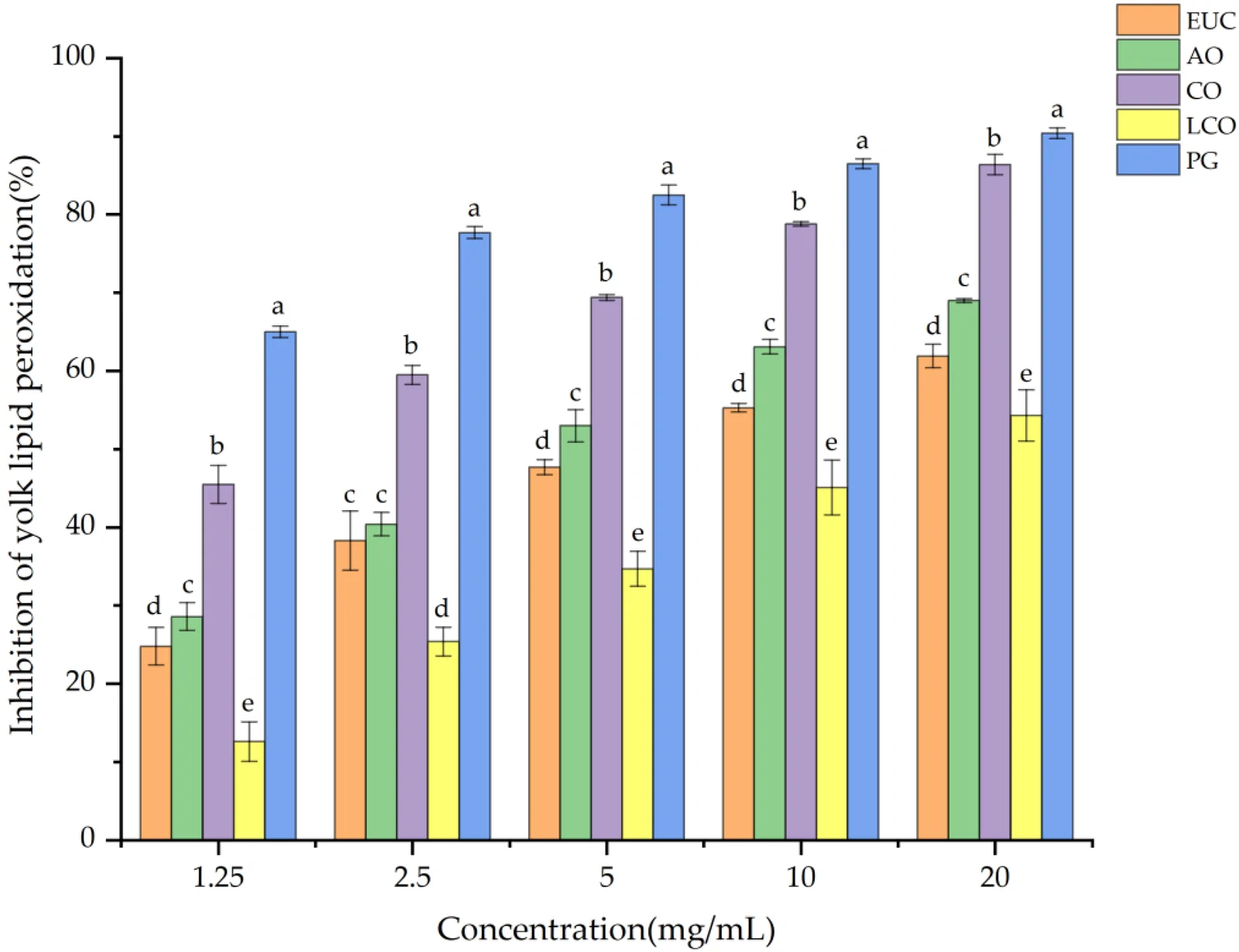
Effect of different concentrations of EOs on yolk lipid peroxidation inhibition. EOs (E) and their different concentrations (C) had significant effects on yolk lipid peroxidation inhibition (*p_E_* < 0.01, *p_C_* < 0.01), and their interaction (E × C) was significant (*p_E_* × *p_C_* < 0.01). Different letters show differences (*p* < 0.05) among EOs. LCO: litsea cubeba oil, CO: cinnamon oil, AO: anise oil, EUC: eucalyptus oil; PG: propyl gallate.

Figure 4 shows the effect of the EO type and the EO concentration on the lipid peroxidation inhibition rate of yolk. EO type (E) and EO concentration (C) had significant effects on the lipid peroxidation inhibition rate of yolk (*p_E_* < 0.01, *p_C_* < 0.01), and the interactions of EO type and concentration (E × C) were significant (*p_E_ × p_C_* < 0.01). The lipid peroxidation inhibition rate of yolk quadratically increased (*p*_Q_ < 0.01) with increasing the concentration of LCO, CO, AO and EUC. In comparing among EOs, the lipid peroxidation inhibition rate of yolk differed (*p* < 0.05) at a concentration of 1.25, 5, 10, 20 mg/mL (PG > CO > AO > EUC > LCO) and at the concentration of 4 mg/mL (CO > AO, EUC > LCO). At the concentration of 2.5 mg/mL, the inhibition rate of PG on yolk lipid peroxidation was 90.41%, and that of CO was 86.42%, reaching 95.59% of PG.

## 3. Discussion

A reduction in antioxidant defense mechanisms and the production of free radicals can lead to oxidative stress, which damages proteins, lipids, and enzymes [16]. Free radicals exist in the body widely, and they are the main cause of lipid peroxidation and the root of oxidative damage to macromolecular substances [17]. Some studies have shown that oxidative stress can cause inflammation and other diseases by producing excess free radicals to mediate oxidative damage to cells [18]. Antioxidants can scavenge free radicals and stop free radical reactions. As a result of the carcinogenicity and hepatotoxicity of synthetic antioxidants (such as BHA and BHT), EOs have attracted increasing attention as green natural antioxidants. Some studies have found that EOs can prevent free radical chain reactions and delay the lipid peroxidation process [6,19,20].

EOs are complex mixtures of different volatile and non-volatile components [21]. Different EOs have different chemical compositions. In the present study, the predominant chemical constituents were citral (geranial and neral) in LCO, (E)-cinnamaldehyde in CO, (E)-anethole in AO, and 1,8-cineole in EUC. Citral and cinnamaldehyde are aldehydes, anethole is a phenylpropanoid, and 1,8-cineole is an oxygenated monoterpene. The reasons for differences in EO composition may be related to plant variety, heredity, maturity stage, culture conditions, and other factors [22].

It has been reported that EOs can reduce or eliminate free radicals, block free radical chain reactions, and delay lipid peroxidation [23]. In our study, the antioxidant activity of four EOs was evaluated through scavenging DPPH free radicals, chelating Fe^2+^, scavenging hydroxyl free radicals, and inhibiting yolk lipid peroxidation. Our study found that the antioxidant activity of EOs is related to the EO concentration and EO type. The DPPH and hydroxyl radical scavenging rate, the Fe^2+^ chelating ability, and the inhibition rate of the lipid peroxidation of yolk were dose-dependent, and they linearly or quadratically increased with an increase in EO concentration, which suggests that the antioxidant activity of EOs varies with concentration. Similarly, Lalami et al. [24] found that the DPPH radical inhibition percentages of cinnamon and clove essential oils presented a dose dependent effect. Other studies have found that the scavenging effect of AO was stronger than BHA and BHT and increased with the increase of concentration [25]. In DPPH radical scavenging experiments, the position of different EOs changes at different concentrations (cross effect). Similar results have been observed in our previous study, where it was found that the antioxidant activity of five citrus EOs increased with an increase in concentration. Additionally, the scavenging effect of bergamot EO was higher than that of lemon EO at low concentrations of 16–80 mg/mL, but the results were the opposite at higher concentrations of 112–144 mg/mL [26]. This is due to the interaction between the type of EO and its concentration. In addition, different EOs had different antioxidant activity. Wei et al. [27] measured the antioxidant activity of 25 EOs and found that cinnamon leaf oil, clove leaf oil, and thyme oil had good antioxidant activity, while sandalwood oil showed poor antioxidant activity. Similarly, Ghazghazi et al. [28] found significant differences in the antioxidant activity of two EOs (*Eucalyptus marginata* L. and *Eucalyptus pauciflora* L. EOs). Another study analyzed the antioxidant activity of LCO extracted in different months, and the results showed that LCO extracted in July had a better hydroxyl radical scavenging rate, and LCO extracted in August had a better DPPH scavenging activity and ferric reducing antioxidant power [29]. In this study, at the same concentration, EUC and CO had good radical hydroxyl scavenging ability, while AO and LCO had poor scavenging ability. This difference may be due to the different chemical compositions of different EOs, and the compositions and contents of EOs may affect their overall antioxidant activity [30].

In addition, the antioxidant activity of EOs is related to their chemical compositions [31]. It has been reported that phenols contribute greatly to the antioxidant capacity of EOs [32]. The phenolic hydroxyl can release hydrogen ions to bind with free radicals and block the oxidative chain reaction [33], and the antioxidant activity of EOs is significantly positively correlated with the phenol content [34]. Mechergui et al. [35] reported that the antioxidant activity of oregano oil mainly depended on the carvall and thymol contents. In addition, fatty acids, esters, ketones, alcohols, and terpenoids have previously been reported to have antioxidant activity [36]. Adefegha et al. [37] showed that the free radical scavenging ability and metal chelating ability of Masson pine EO may be attributed to phenolic monoterpenes and oxygenated monoterpenes in EOs. Some studies have shown that the antioxidant activity of different antioxidants mainly depends on their stability after absorbing lone pair electrons, and phenols > aldehydes > alcohols [38]. The four EOs showed different antioxidant activity, which may have been caused by the different chemical components of different EOs. The active components of EOs are complex and diverse, and whether the antioxidant activity of different active components are based on synergistic, antagonistic, or additive effects needs to be further studied.

Various antioxidant activity assays have been used to evaluate the antioxidant capacity of EOs in vitro, and EOs showed different antioxidant activity using different assays. In our study, we evaluated the antioxidant activity of EOs using four methods: scavenging DPPH radical, chelating Fe^2+^, scavenging hydroxyl radical, and inhibiting yolk lipid peroxidation. DPPH free radical is a stable radical that is widely used as a tool to evaluate the free radical scavenging activity of EOs [39]. The ability of EOs to scavenge DPPH free radicals may be related to their ability to provide hydrogen atoms. EOs can provide hydrogen atoms to DPPH radicals to form stable end products (DPPH-H), which will not further trigger or aggravate lipid oxidation [40]. It has been found that monoterpenes, such as citral, carvone, laurene, and γ-terpinene, have strong DPPH radical scavenging activity [41]. The scavenging ability of EOs against DPPH free radicals is related to their content of unsaturated hydrocarbon. LCO had the strongest scavenging ability against DPPH free radicals among the four EOs, which may be due to the effects of citral, the main component of LCO. The accumulation of Fe^2+^ in cells can induce the production of free radicals and malondialdehyde [37,42]. EOs can chelate with metal ions to form stable complexes that inhibit the formation of metal-induced free radicals. Some phenols in EOs can effectively chelate ferrous ions [43]. Singh et al. [44] found that the chelating activity of EOs of *Zanthoxylum armatum* DC was significantly higher than that of BHA and BHT, which may be attributed to the phenolic compounds contained in EOs that can chelate metal ions. In our study, EUC showed a good Fe^2+^ chelating ability and increased in a dose-dependent manner, which may be due to the large number of compounds in the oil and their possible affinity for Fe^2+^. Hydroxyl free radical is a kind of highly active oxidant that can directly damage various biofilms and lead to many diseases [45]. Hydroxyl free radicals can extract electrons from polyunsaturated fatty acids and produce lipid radicals, which can trigger lipid peroxidation. In addition, hydroxyl free radicals are highly reactive products produced via the decomposition of hydrogen peroxide in the presence of Fe^2+^ and other metals, which can aggravate oxidative stress in the body, lead to immune system dysfunction, and affect the digestion, absorption, and transformation of nutrients [46]. Thus, a reduction in hydroxyl radicals can reduce lipid peroxidation and protect the immune system. In our study, all four EOs could remove hydroxyl radicals, among which EUC has the best effect. The scavenging effects of EOs on hydroxyl free radicals can not only act on the non-chain process before the oxidation reaction chain to remove the hydroxyl radicals, starting the chain reaction, but also remove the free radicals in the chain extension process, producing a dual-antioxidant effect. Lipid peroxidation is considered to be an important mechanism of cell damage under oxidative stress and is involved in the pathological processes of many diseases [47]. Previous studies have shown that EOs can effectively inhibit lipid peroxidation. Gargouri et al. [48] found that Henna EO showed very high potential for scavenging free radicals and inhibiting lipid peroxidation, which could be used to combat the oxidative stress generated by Raji cells. Alizadeh et al. [49] speculated that the EO of *Zeilan cinnamomum* L. had a strong free radical scavenging ability and the potential to inhibit lipid oxidation reactions, which was consistent with the results of our experiment. In our study, CO showed the best inhibition of lipid peroxidation of yolk, which may be attributed to the fact that its main component, 1,8 eucalyptol, is a monoterpenoid. It has been found that monoterpene hydrocarbon, γ-terpinene, retards the peroxidation of linoleic acid because its chaincarrying peroxyls are HOO• radicals, which react rapidly with linoleylperoxyl radicals [50]. In our study, the four EOs showed different antioxidant activity in different antioxidant tests. EUC showed good hydroxyl radical scavenging ability and Fe^2+^ chelating ability, but poor DPPH radical scavenging ability and yolk lipid peroxidation inhibition. Similarly, Bi et al. [51] found that the EOs from Nandina domestica fruits exhibited significant scavenging activity against DPPH free radical and ATBS free radical, moderate scavenging activity against superoxide anion free radical, and low activity against metal chelating power. This difference may be due to the fact that the total antioxidant activity of EOs is the result of a combination of one or several pathways. In our study, EUC had the best hydroxyl radical scavenging ability and chelating Fe^2+^ ability, and CO had better yolk lipid peroxidation inhibition ability and DPPH and hydroxyl radical scavenging ability. Therefore, EUC and CO have good antioxidant activity and have the potential to be used in practical production as a new green antioxidant.

In summary, our results show that the four EOs possessed significant antioxidant activity, but their antioxidant capacity varied with EO type and concentration. In addition, the different antioxidant abilities of EOs may be due to the different chemical compositions of EOs. Combined with the results of the four antioxidant experiments, it is speculated that the total antioxidant activity of EOs may comprise different activities including scavenging free radicals, chelating Fe^2+^, and inhibiting lipid peroxidation etc.

## 4. Materials and Methods

### 4.1. Chemicals and Reagents

Butylated hydroxyanisole was purchased from Beijing Solarbio Science and Technology Co., Ltd. (Beijing, China). DPPH was purchased from Sigma–Aldrich (Shanghai, China) and Sigma–Aldrich (St. Louis, MO, USA). Propyl gallat, vitamin C, Acido salicilico, and 2-thiobarbituric acid (TBA) were purchased from Sinopharm Chemical Reagent Co., Ltd. (Shanghai, China).

### 4.2. Essential Oils

LCO (purity 80%), CO (purity 80%), AO (purity 87%), and EUC (purity 80%) were purchased from Nanjing Vincero International Trade Co., Ltd. (Nanjing, China).

### 4.3. Gas Chromatography-Mass Spectrometry (GC-MS) Analysis

The EO samples were diluted 1:100 with hexane and then injected into an Rxi-5Sil MS (Restek, Bellefonte, Newcastle, PA, USA, 30 m × 0.25 mm × 0.25 μm) column via the GC-MS injection system. The column temperature was initially set at 50 °C and held for 5 min, then increased up to 320 °C at a rate of 6 °C/min for 5 min and solvent delay for 3 min. Helium was used as a carrier with a shunt ratio of 10:1 and a flow rate of 1 mL/min. Total ion flow chromatography (TIC) was obtained by analyzing the samples. The data were collected using the GC-MS solution 2.6 software and screened and matched with NIST and other dedicated standard spectrum libraries to identify each component of the EOs. Compound identification was based primarily on comparison of the acquired mass spectra with the NIST and other dedicated mass spectral libraries. Retention index (RI) were obtained from established databases and published literature for low-polarity stationary phases comparable to the Rxi-5Sil MS column. Detailed NIST library-matching information and row-level documentation of the RI sources and reporting rationale are provided in Supplementary Tables S1 and S2, respectively. Finally, the relative contents of the components were calculated using peak-area normalization.

### 4.4. Antioxidant Activity

#### 4.4.1. Experimental Design

The antioxidant activity of the four EOs at different concentrations was evaluated by measuring DPPH free radical scavenging ability, Fe^2+^ chelating ability, hydroxyl radical scavenging ability, and yolk lipid peroxidation inhibition. The experiment was a completed randomized design with 4 EOs (LCO, CO, AO, and EUC) × 5 concentrations of factorial arrangements of treatments. The concentration of each EO was 2.5, 10, 20, 40, and 80 mg/mL in the DPPH measurements; 2, 6, 8, 10, and 12 mg/mL in the Fe^2+^ chelating ability assay; 1, 4, 8, 16, and 32 mg/mL in the hydroxyl radical scavenging ability assay; and 1.25, 2.5, 5, 10, and 20 mg/mL in the yolk lipid peroxidation inhibition assay. Butylated hydroxyanisole (BHA), propyl gallate (PG), and vitamin C (VC), as strong antioxidants, were used as controls, respectively.

#### 4.4.2. DPPH Radical Scavenging Ability Assay

The DPPH free radical scavenging activity was determined according to a previously described method [52]. LCO, CO, AO, EUC, and BHA were diluted with anhydrous ethanol to 2.5, 10, 20, 40, and 80 mg/mL, respectively. A 2 mL EOs diluent and 2 mL 0.2 mmol/L DPPH ethanol solution were taken to prepare 4 mL of the tested solution in a tube. After shaking, the solution was kept away from light at room temperature for 30 min. Next, 200 μL of the tested solution was added to a 96-well microtiter plate. The absorbance was measured using an automatic microplate reader at 517 nm, and the clearance rate was calculated. There were 3 parallel groups in each group. The DPPH free radical scavenging rate was calculated according to Formula (1). A_1_ is the absorbance of the ethanol solution of DPPH mixed with the diluent of plant EOs, A_0_ is the absorbance of the DPPH ethanol solution mixed with anhydrous ethanol, and A_2_ is the absorbance of the plant EOs diluent mixed with absolute ethanol.

DPPH radical scavenging rate = [A_0_ − (A_1_ − A_2_)]/A_0_ × 100% (1)

#### 4.4.3. Fe^2+^ Chelating Ability Assay

The Fe^2+^ chelating ability was determined as described by Oboh et al. [53]. LCO, CO, AO, EUC, and strong antioxidant (VC) were diluted with anhydrous ethanol to 2, 6, 8, 10, and 12 mg/mL, respectively. A total of 1 mL of the diluted sample solution was taken, and 0.1 mL of 2 mmol/L FeCl_2_ and 0.3 mL of 5 mmol/L phenanthrazine were successively added to the clean test tube. Then, 2.6 mL of distilled water was added to prepare 4 mL of the tested solution, and the solution was left at room temperature for 20 min. A total of 200 μL of the tested solution was added to a 96-well microtiter plate. The absorbance was measured using an automatic microplate reader at 517 nm, and the clearance rate was calculated. There were 3 parallel groups in each group. The chelating capacity of Fe^2+^ was calculated according to Formula (2). A_1_ is the absorbance of the tested solution added to EOs, A_0_ is the absorbance of absolute ethanol sample solution without EOs, and A_2_ is the absorbance of distilled water without phenanthrazine.

Fe^2+^ chelating capacity (%) = [A_0_ − (A_1_ − A_2_)/A_0_] × 100% (2)

#### 4.4.4. Hydroxyl Radical Scavenging Ability Assay

The hydroxyl radical scavenging ability was measured according to the method of Radünz [54] with some modifications. LCO, CO, AO, EUC, and strong antioxidant (VC) were diluted with anhydrous ethanol to 1, 4, 8, 16, and 32 mg/mL, respectively. A total of 1 mL of essential oil diluent was taken from each, and 1 mL of a 2 mmol/L FeSO_4_, 1 mL of a 6 mmol/L salicylic acid, and 1 mL of a 2 mmol/L H_2_O_2_ were added to prepare 4 mL of the tested sample solution. The solution was mixed well and left for 30 min at room temperature. A total of 200 μL of the tested solution was added to a 96-well microtiter plate. The absorbance was measured using an automatic microplate reader at 510 nm and the clearance rate was calculated. There were 3 parallel groups in each group. The hydroxyl radical scavenging rate was calculated according to Formula (3). A_1_ is the absorbance of the tested solution added to EOs; A_0_ is the absorbance of absolute ethanol sample solution without EOs; A_2_ is the absorbance of distilled water without H_2_O_2_.

The hydroxyl radical scavenging rate (%)= [A_0_ − (A_1_ − A_2_)]/A_0_ × 100% (3)

#### 4.4.5. Yolk Lipid Peroxidation Inhibition Assay

The yolk lipid peroxidation inhibition rate was determined according to a previously described method [55]. The yolk of fresh eggs was put into a 50 mL beaker, the same volume of phosphate buffer was added, the sealing film was fastened, and the solution was stored at −4 °C. Four EOs and PG were diluted with absolute ethanol to 1.25, 2.5, 5, 10, and 20 mg/mL sample dilutions. A total of 0.5 mL of egg yolk stock solution was taken and 12 mL of phosphate buffer was added to prepare a 1:25 egg yolk suspension. A total of 200 μL of the yolk suspension was taken, a 100 μL EO sample diluent was added, and then 200 μL of a 25 mmol/L FeSO_4_ was added and supplemented to 1 mL with phosphate buffer. After shaking at 37 °C for 30 min, 250 μL of a 20% trichloroacetic acid was added, 3500 r/min centrifugation was performed for 10 min until clarification, the supernatant was absorbed, and 250 μL of a 0.8% TBA was added. A total of 1.5 mL of the sample solution was prepared to be tested and reacted in a boiling water bath for 15 min. A total of 200 μL of the tested solution was added to a 96-well microtiter plate. The absorbance was measured using an automatic microplate reader at 510 nm, and the clearance rate was calculated. There were 3 parallel groups in each group. The yolk lipid peroxidation inhibition rate was calculated according to Formula (4). A_1_ is the absorbance of the tested solution added to EOs; A_0_ is the absorbance of absolute ethanol sample solution without EOs; A_2_ is the absorbance of the tested solution without yolk.

The yolk lipid peroxidation inhibition rate (%) = [A_0_ − (A_1_ − A_2_)/A_0_] × 100% (4)

## 5. Statistical Analysis

Data for antioxidant activity were analyzed using the SAS 9.4 mixing procedure with a model including EO type, EO concentration, and their interactions as fixed effects and three replications of the experiment as random effects. The effects of increasing the EO concentration were examined through linear and quadratic orthogonal contrasts using the CONTRAST statement of SAS. Differences were declared significant at *p* ≤ 0.05. The graphs were drawn using the Origin 2021 software (Northampton, MA, USA).

## 6. Conclusions

The major constituents of LCO, CO, AO, and EUC were citral (geranial and neral), (E)-cinnamaldehyde, (E)-anethole, and 1,8-cineole, respectively. The four EOs effectively scavenged DPPH, chelated Fe^2+^, scavenged hydroxyl radicals, and inhibited lipid peroxidation in a dose-dependent manner. Compared with the other EOs, LCO had the strongest DPPH radical-scavenging ability; EUC had the greatest Fe^2+^-chelating and hydroxyl radical-scavenging activities; and CO had the highest yolk lipid-peroxidation-inhibitory activity. It is speculated that the total antioxidant activity of EOs might result from the joint actions of different antioxidant capacities.

## Figures and Tables

**Table 1:** GC-MS analysis of chemical profiles and relative abundances of four essential oils.

No.	RT (min)	RI	Compound	LCO (%)	CO (%)	AO (%)	EUC (%)	ID
1	2.02	n.a.	Unidentified EUC-1 (best MS match: 1-methylhexyl hydroperoxide)	-	-	-	0.03	C
2	6.70	936	α-Pinene	1.46	-	0.98	0.30	A; EUC B
3	7.25	950	Camphene	0.35	-	-	0.16	A
4	7.75	963	Benzaldehyde	-	1.06	-	-	A
5	8.08	973	Sabinene	1.08	-	-	2.19	A
6	8.19	978	β-Pinene	1.13	-	-	1.34	A
7	8.57	986	6-Methyl-5-hepten-2-one	1.80	-	-	-	A
8	8.70	989	β-Myrcene	0.71	-	-	4.58	A
9	9.17	1004	α-Phellandrene	-	-	0.48	1.97	A
10	9.24	1011	3-Carene (tentative)	-	-	0.37	-	B
11	9.53	1018	4-Carene (tentative)	-	-	-	5.35	B
12	9.83	n.a.	Cymene isomer (tentative; m-/p-cymene unresolved)	-	-	-	1.81	B
13	9.90	1030	Limonene	12.39	-	2.20	-	A
14	10.00	1032	1,8-Cineole (eucalyptol)	-	-	0.70	81.79	A
15	10.82	1060	γ-Terpinene	-	-	-	0.19	A
16	12.22	1099	Linalool	2.08	-	2.21	-	A
17	13.29	n.a.	Unidentified LCO-9 (best MS match: 3,3,5-trimethyl-1,4-hexadiene)	0.26	-	-	-	C
18	13.33	n.a.	Unidentified EUC-12 (best MS match: 2,3,5-trimethyl-1,3-hexadiene)	-	-	-	0.25	C
19	13.54	1154	Citronellal	2.47	-	-	-	A
20	13.54	n.a.	Unidentified EUC-13 (best MS match: 3,3,5-trimethylcyclohexyl methacrylate)	-	-	-	0.05	C
21	13.79	n.a.	2,4,6-Trimethylcyclohex-3-ene-1-carbaldehyde (tentative)	1.32	-	-	-	B
22	13.79	1163	3-Phenylpropanal (hydrocinnamaldehyde)	-	0.74	-	-	A
23	14.20	1166	endo-Borneol	0.10	-	-	-	A
24	14.29	n.a.	Unresolved oxygenated monoterpene LCO-13 (best MS match: trans-4,5-epoxycarane)	2.62	-	-	-	C
25	14.30	1177	Terpinen-4-ol	-	-	0.30	-	A
26	14.75	1196	Estragole	-	-	6.15	-	A
27	14.77	1190	α-Terpineol	0.78	-	-	-	A
28	15.29	1215	(Z)-Cinnamaldehyde (cis; tentative)	-	0.39	-	-	B
29	15.64	1229	Nerol	0.72	-	-	-	A
30	15.89	1242	Neral (β-citral)	27.97	-	-	-	A
31	16.12	1249	(Z)-Anethole (cis; tentative)	-	-	0.45	-	B
32	16.18	1254	Piperitone	0.13	-	-	-	A
33	16.26	1252	p-Anisaldehyde	-	-	1.06	-	A
34	16.32	1255	Geraniol	1.93	-	-	-	A
35	16.66	1270	Geranial (α-citral)	35.58	-	-	-	A
36	16.88	1271	(E)-Cinnamaldehyde (trans)	-	84.86	-	-	A
37	17.09	n.a.	Unidentified LCO-20 (best MS match: limonene oxide)	0.32	-	-	-	C
38	17.16	1285	(E)-Anethole (trans)	-	-	78.51	-	B
39	18.10	n.a.	Unidentified LCO-21 (best MS match: 2,7-dimethyl-2,7-octanediol; literature-reported candidate)	0.24	-	-	-	C
40	18.70	n.a.	Unidentified LCO-22 (best MS match: 2,4,4,7-tetramethyl-5,7-octadien-3-ol)	0.21	-	-	-	C
41	18.92	n.a.	Unidentified LCO-23 (best MS match: 6-hepten-3-ol)	0.39	-	-	-	C
42	18.96	1376	α-Copaene	-	0.57	-	-	B
43	19.09	n.a.	Unidentified AO-12 (best MS match: 2-phenyloxiranemethanol)	-	-	0.32	-	C
44	19.28	1390	β-Elemene	0.33	-	-	-	A
45	19.79	1415	cis-α-Bergamotene (tentative)	-	-	0.18	-	B
46	19.93	1420	(E)-Caryophyllene (β-caryophyllene)	2.37	0.18	0.80	-	A; CO B
47	20.22	1435	trans-α-Bergamotene	-	0.12	0.92	-	B
48	20.38	1432	Coumarin (tentative)	-	6.43	-	-	B
49	20.52	1448	Cinnamyl acetate	-	1.46	-	-	A
50	20.79	1460	Alloaromadendrene	-	0.19	-	-	A
51	21.11	1476	γ-Muurolene	-	0.22	-	-	A
52	21.54	n.a.	Unidentified AO-16 (best MS match: Germacrene B; retention conflict)	-	-	0.24	-	C
53	21.61	1482	α-Amorphene	-	0.12	-	-	B
54	21.80	1508	β-Bisabolene	-	0.38	0.20	-	A
55	21.90	n.a.	Unidentified CO-14 (best MS match: β-copaene; retention conflict)	-	0.17	-	-	C
56	22.01	1523	δ-Cadinene	-	0.35	-	-	A
57	22.18	n.a.	Unidentified CO-16 (best MS match: 4,8,11,11-tetramethyltricyclo[7.2.0.0(3,8)]undec-4-ene)	-	0.16	-	-	C
58	22.27	1505	2-Methoxycinnamaldehyde (tentative)	-	1.76	-	-	B
59	22.92	1561	(E)-Nerolidol	-	0.34	0.24	-	A
60	23.30	1581	Caryophyllene oxide	0.56	0.35	-	-	A
61	24.76	1652	α-Cadinol	-	-	0.23	-	B
62	25.13	1676	Foeniculin	-	-	3.47	-	B
63	30.76	n.a.	Unidentified CO-20 (best MS match: β-selinene; retention conflict)	-	0.15	-	-	C
64	31.51	2201	trans-Geranylgeraniol	0.10	-	-	-	B
65	31.63	n.a.	Unidentified LCO-28 (best MS match: isoaromadendrene epoxide)	0.20	-	-	-	C
66	32.76	n.a.	Unidentified LCO-29 (best MS match: neric acid)	0.22	-	-	-	C
67	32.89	n.a.	Unidentified LCO-30	0.16	-	-	-	C

Note: LCO, Litsea cubeba oil; CO, cinnamon oil; AO, anise oil; EUC, eucalyptus oil; RT, retention time. A hyphen indicates that the corresponding peak was not detected. Relative contents are peak-area percentages; totals are 99.98% (LCO), 100.00% (CO), 100.01% (AO), and 100.01% (EUC). RT, retention time were obtained from separate injections of the four essential oils. RI, retention index denotes reference retention indices obtained from established databases and published literature for stationary phases comparable to the Rxi-5Sil MS column. These values were used only as supporting evidence to assess the plausibility of the library-based assignments and were not experimentally determined in the present study. ID, Identification confidence: A, high EI-MS agreement with plausible retention behavior; B, tentative isomer/common-name assignment or moderate evidence; C, unidentified because of low similarity and/or retention conflict.

## Data Availability

Data present in this study are available on request from the corresponding author.

## Supplementary Materials

The following supporting information can be downloaded at https://www.mdpi.com/article/10.3390/molecules28135051/s1. Table S1. Peak-level NIST11 library matching information for the four original GC-MS reports; Table S2. Row-level provenance of the RI values reported in Table 1 and the corresponding identification assessments.

## Sample Availability

Samples of the compounds are available from the authors.
