# Expression of Concern: Liu et al. Comparison of Chemical Compositions and Antioxidant Activity of Essential Oils from Litsea Cubeba, Cinnamon, Anise, and Eucalyptus. *Molecules* 2023, *28*, 5051

**DOI:** 10.3390/molecules31162802

**Published:** 2026-08-12

**Authors:** Molecules Editorial Office

**Affiliations:** MDPI, Grosspeteranlage 5, 4052 Basel, Switzerland; molecules@mdpi.com

With this notice, the Molecules Editorial Office wishes to alert readers to concerns related to this article [1]. Following publication, concerns were raised regarding inconsistencies and errors in the chemical analysis presented in this paper.

The authors have been contacted for further clarification. The investigation is being coordinated by the Editorial Office with the involvement of the Editorial Board. Once this process is complete, the Editorial Office will provide an update on this situation and announce any post-publication modification deemed to be necessary, as per MDPI’s Updating Published Papers policy.

## References

[1] Liu S., Zhao C., Cao Y., Li Y., Zhang Z., Nie D., Tang W., Li Y. (2023). Comparison of Chemical Compositions and Antioxidant Activity of Essential Oils from Litsea Cubeba, Cinnamon, Anise, and Eucalyptus. Molecules.

