# Correction: Liu et al. Comparison of Chemical Compositions and Antioxidant Activity of Essential Oils from Litsea Cubeba, Cinnamon, Anise, and Eucalyptus. *Molecules* 2023, *28*, 5051

**DOI:** 10.3390/molecules31193473

**Published:** 2026-09-29

**Authors:** Shutian Liu, Chen Zhao, Yuwei Cao, Yan Li, Zhuo Zhang, Dechao Nie, Weixuan Tang, Yanling Li

**Affiliations:** Animal Science and Technology College, Beijing University of Agriculture, No. 7 Beinong Road, Changping, Beijing 102206, China

## Error in Table 1

In the original publication [1], the RI values shown in Table 1 were taken from NIST library-search records associated with candidate compounds and were presented as though they were experimental RIs for the four chromatograms. The original analytical reports did not contain a same-run n-alkane calibration; therefore, those library values were not RIs measured under the chromatographic conditions of this study. In addition, some library candidates were reported with more structural or stereochemical specificity than supported by the EI-MS and non-chiral column, and summary ‘Others’ values were used to bring the displayed totals to 100%.

The authors rechecked every peak against the four original GC-MS reports and re-evaluated the library candidates using spectral agreement, retention behavior, and independent literature/database RI records for comparable 5%-phenyl low-polarity phases. The corrected Table 1 retains the measured RT and peak-area percentage, reports literature/database RI values, and states an identification-confidence class. Unsupported enantiomeric labels were removed, ambiguous isomers were reported conservatively, and peaks with low similarity or material retention conflicts were designated tentative or unidentified. The RI values reported in the original article were not reused as reference evidence in this re-evaluation. Supplementary Table S1 preserves the original NIST11 search records, while Supplementary Table S2 clearly distinguishes the original literature/database evidence from the RI values selected for reporting in the revised Table 1 and the corresponding identification assessments.

The corrected Table 1 appears below.

## Text Corrections

Table 1 was revised with changes to compound assignments, peak counts, relative abundances, and identification confidence; unsupported assignments were downgraded to tentative or unidentified, and the summary ‘Others’ category was removed. Consequently, the Abstract, Section 2.1, Discussion, Methods, Conclusions, and Supplementary Materials were updated so that the reported major constituents, percentages, and identification terminology were consistent with the corrected Table 1.

The corresponding text has been updated as follows.

**Abstract:** The purpose of this study was to compare the antioxidant activity of litsea cubeba oil (LCO), cinnamon oil (CO), anise oil (AO), and eucalyptus oil (EUC) in vitro. The chemical compositions of the essential oils (EOs) were analyzed using gas chromatography-mass spectrometry (GC-MS). The antioxidant activity of the four EOs was evaluated through scavenging DPPH free radicals, chelating Fe^2+^, scavenging hydroxyl free radicals, and inhibiting yolk lipid peroxidation. The revised compositional analysis showed that the major constituents of LCO, CO, AO, and EUC were citral (geranial plus neral, 63.55%), (E)-cinnamaldehyde (84.86%), (E)-anethole (78.51%), and 1,8-cineole (81.79%), respectively. The four EOs all had certain antioxidant activity. The ability to scavenge DPPH radical was ranked in the order of LCO > CO > AO > EUC. The hydroxyl radical scavenging ability was ranked in the order of EUC > CO > LCO > AO. The chelating Fe^2+^ capacity was ranked in the order of EUC > AO > CO > LCO. The yolk lipid peroxidation inhibition ability was ranked in the order of CO > AO > EUC > LCO. In different antioxidant activity assays, the antioxidant activity of the EOs was different. It was speculated that the total antioxidant activity of an EO may be the result of the joint action of different antioxidant capacities.

**Section 2.1. Chemical Compositions of Essential Oils:** The chemical compositions of the four EOs are shown in Table 1. 30, 20, 20, and 13 peaks were detected in LCO, CO, AO, and EUC, respectively. LCO was dominated by geranial (35.58%), neral (27.97%), and limonene (12.39%); geranial and neral together accounted for 63.55% citral. An unresolved oxygenated monoterpene peak accounted for 2.62%. The major constituents of CO were (E)-cinnamaldehyde (84.86%), tentative coumarin (6.43%), tentative 2-methoxycinnamaldehyde (1.76%), and cinnamyl acetate (1.46%). AO contained (E)-anethole (78.51%), estragole (6.15%), tentative foeniculin (3.47%), linalool (2.21%), and limonene (2.20%). EUC contained 1,8-cineole (81.79%), tentative 4-carene (5.35%), β-myrcene (4.58%), sabinene (2.19%), and α-phellandrene (1.97%). Identification confidence (ID) and unresolved assignments are indicated in Table 1.

**Section 3. Discussion:** EOs are complex mixtures of different volatile and non-volatile components [21]. Different EOs have different chemical compositions. In the present study, the predominant chemical constituents were citral (geranial and neral) in LCO, (E)-cinnamaldehyde in CO, (E)-anethole in AO, and 1,8-cineole in EUC. Citral and cinnamaldehyde are aldehydes, anethole is a phenylpropanoid, and 1,8-cineole is an oxygenated monoterpene. The reasons for differences in EO composition may be related to plant variety, heredity, maturity stage, culture conditions, and other factors [22].

**Section 4.3. Gas Chromatography-Mass Spectrometry (GC-MS) Analysis:** The EO samples were diluted 1:100 with hexane and then injected into an Rxi-5Sil MS (Restek, Bellefonte, Newcastle, PA, USA, 30 m × 0.25 mm × 0.25 μm) column via the GC-MS injection system. The column temperature was initially set at 50 °C and held for 5 min, then increased up to 320 °C at a rate of 6 °C/min for 5 min and solvent delay for 3 min. Helium was used as a carrier with a shunt ratio of 10:1 and a flow rate of 1 mL/min. Total ion flow chromatography (TIC) was obtained by analyzing the samples. The data were collected using the GC-MS solution 2.6 software and screened and matched with NIST and other dedicated standard spectrum libraries to identify each component of the EOs. Compound identification was based primarily on comparison of the acquired mass spectra with the NIST and other dedicated mass spectral libraries. Retention index (RI) were obtained from established databases and published literature for low-polarity stationary phases comparable to the Rxi-5Sil MS column. Detailed NIST library-matching information and row-level documentation of the RI sources and reporting rationale are provided in Supplementary Tables S1 and S2, respectively. Finally, the relative contents of the components were calculated using peak-area normalization.

**Section 6. Conclusions:** The major constituents of LCO, CO, AO, and EUC were citral (geranial and neral), (E)-cinnamaldehyde, (E)-anethole, and 1,8-cineole, respectively. The four EOs effectively scavenged DPPH, chelated Fe^2+^, scavenged hydroxyl radicals, and inhibited lipid peroxidation in a dose-dependent manner. Compared with the other EOs, LCO had the strongest DPPH radical-scavenging ability; EUC had the greatest Fe^2+^-chelating and hydroxyl radical-scavenging activities; and CO had the highest yolk lipid-peroxidation-inhibitory activity. It is speculated that the total antioxidant activity of EOs might result from the joint actions of different antioxidant capacities.

**Supplementary Materials:** The following supporting information can be downloaded at https://www.mdpi.com/article/10.3390/molecules28135051/s1. Table S1. Peak-level NIST11 library matching information for the four original GC-MS reports; Table S2. Row-level provenance of the RI values reported in Table 1 and the corresponding identification assessments.

The authors state that the scientific conclusions are unaffected. This correction was approved by the Academic Editor. The original publication has also been updated.

## Figures and Tables

**Table 1:** GC-MS analysis of chemical profiles and relative abundances of four essential oils.

No.	RT (min)	RI	Compound	LCO (%)	CO (%)	AO (%)	EUC (%)	ID
1	2.02	n.a.	Unidentified EUC-1 (best MS match: 1-methylhexyl hydroperoxide)	-	-	-	0.03	C
2	6.70	936	α-Pinene	1.46	-	0.98	0.30	A; EUC B
3	7.25	950	Camphene	0.35	-	-	0.16	A
4	7.75	963	Benzaldehyde	-	1.06	-	-	A
5	8.08	973	Sabinene	1.08	-	-	2.19	A
6	8.19	978	β-Pinene	1.13	-	-	1.34	A
7	8.57	986	6-Methyl-5-hepten-2-one	1.80	-	-	-	A
8	8.70	989	β-Myrcene	0.71	-	-	4.58	A
9	9.17	1004	α-Phellandrene	-	-	0.48	1.97	A
10	9.24	1011	3-Carene (tentative)	-	-	0.37	-	B
11	9.53	1018	4-Carene (tentative)	-	-	-	5.35	B
12	9.83	n.a.	Cymene isomer (tentative; m-/p-cymene unresolved)	-	-	-	1.81	B
13	9.90	1030	Limonene	12.39	-	2.20	-	A
14	10.00	1032	1,8-Cineole (eucalyptol)	-	-	0.70	81.79	A
15	10.82	1060	γ-Terpinene	-	-	-	0.19	A
16	12.22	1099	Linalool	2.08	-	2.21	-	A
17	13.29	n.a.	Unidentified LCO-9 (best MS match: 3,3,5-trimethyl-1,4-hexadiene)	0.26	-	-	-	C
18	13.33	n.a.	Unidentified EUC-12 (best MS match: 2,3,5-trimethyl-1,3-hexadiene)	-	-	-	0.25	C
19	13.54	1154	Citronellal	2.47	-	-	-	A
20	13.54	n.a.	Unidentified EUC-13 (best MS match: 3,3,5-trimethylcyclohexyl methacrylate)	-	-	-	0.05	C
21	13.79	n.a.	2,4,6-Trimethylcyclohex-3-ene-1-carbaldehyde (tentative)	1.32	-	-	-	B
22	13.79	1163	3-Phenylpropanal (hydrocinnamaldehyde)	-	0.74	-	-	A
23	14.20	1166	endo-Borneol	0.10	-	-	-	A
24	14.29	n.a.	Unresolved oxygenated monoterpene LCO-13 (best MS match: trans-4,5-epoxycarane)	2.62	-	-	-	C
25	14.30	1177	Terpinen-4-ol	-	-	0.30	-	A
26	14.75	1196	Estragole	-	-	6.15	-	A
27	14.77	1190	α-Terpineol	0.78	-	-	-	A
28	15.29	1215	(Z)-Cinnamaldehyde (cis; tentative)	-	0.39	-	-	B
29	15.64	1229	Nerol	0.72	-	-	-	A
30	15.89	1242	Neral (β-citral)	27.97	-	-	-	A
31	16.12	1249	(Z)-Anethole (cis; tentative)	-	-	0.45	-	B
32	16.18	1254	Piperitone	0.13	-	-	-	A
33	16.26	1252	p-Anisaldehyde	-	-	1.06	-	A
34	16.32	1255	Geraniol	1.93	-	-	-	A
35	16.66	1270	Geranial (α-citral)	35.58	-	-	-	A
36	16.88	1271	(E)-Cinnamaldehyde (trans)	-	84.86	-	-	A
37	17.09	n.a.	Unidentified LCO-20 (best MS match: limonene oxide)	0.32	-	-	-	C
38	17.16	1285	(E)-Anethole (trans)	-	-	78.51	-	B
39	18.10	n.a.	Unidentified LCO-21 (best MS match: 2,7-dimethyl-2,7-octanediol; literature-reported candidate)	0.24	-	-	-	C
40	18.70	n.a.	Unidentified LCO-22 (best MS match: 2,4,4,7-tetramethyl-5,7-octadien-3-ol)	0.21	-	-	-	C
41	18.92	n.a.	Unidentified LCO-23 (best MS match: 6-hepten-3-ol)	0.39	-	-	-	C
42	18.96	1376	α-Copaene	-	0.57	-	-	B
43	19.09	n.a.	Unidentified AO-12 (best MS match: 2-phenyloxiranemethanol)	-	-	0.32	-	C
44	19.28	1390	β-Elemene	0.33	-	-	-	A
45	19.79	1415	cis-α-Bergamotene (tentative)	-	-	0.18	-	B
46	19.93	1420	(E)-Caryophyllene (β-caryophyllene)	2.37	0.18	0.80	-	A; CO B
47	20.22	1435	trans-α-Bergamotene	-	0.12	0.92	-	B
48	20.38	1432	Coumarin (tentative)	-	6.43	-	-	B
49	20.52	1448	Cinnamyl acetate	-	1.46	-	-	A
50	20.79	1460	Alloaromadendrene	-	0.19	-	-	A
51	21.11	1476	γ-Muurolene	-	0.22	-	-	A
52	21.54	n.a.	Unidentified AO-16 (best MS match: Germacrene B; retention conflict)	-	-	0.24	-	C
53	21.61	1482	α-Amorphene	-	0.12	-	-	B
54	21.80	1508	β-Bisabolene	-	0.38	0.20	-	A
55	21.90	n.a.	Unidentified CO-14 (best MS match: β-copaene; retention conflict)	-	0.17	-	-	C
56	22.01	1523	δ-Cadinene	-	0.35	-	-	A
57	22.18	n.a.	Unidentified CO-16 (best MS match: 4,8,11,11-tetramethyltricyclo[7.2.0.0(3,8)]undec-4-ene)	-	0.16	-	-	C
58	22.27	1505	2-Methoxycinnamaldehyde (tentative)	-	1.76	-	-	B
59	22.92	1561	(E)-Nerolidol	-	0.34	0.24	-	A
60	23.30	1581	Caryophyllene oxide	0.56	0.35	-	-	A
61	24.76	1652	α-Cadinol	-	-	0.23	-	B
62	25.13	1676	Foeniculin	-	-	3.47	-	B
63	30.76	n.a.	Unidentified CO-20 (best MS match: β-selinene; retention conflict)	-	0.15	-	-	C
64	31.51	2201	trans-Geranylgeraniol	0.10	-	-	-	B
65	31.63	n.a.	Unidentified LCO-28 (best MS match: isoaromadendrene epoxide)	0.20	-	-	-	C
66	32.76	n.a.	Unidentified LCO-29 (best MS match: neric acid)	0.22	-	-	-	C
67	32.89	n.a.	Unidentified LCO-30	0.16	-	-	-	C

Note: LCO, Litsea cubeba oil; CO, cinnamon oil; AO, anise oil; EUC, eucalyptus oil; RT, retention time. A hyphen indicates that the corresponding peak was not detected. Relative contents are peak-area percentages; totals are 99.98% (LCO), 100.00% (CO), 100.01% (AO), and 100.01% (EUC). RT, retention time were obtained from separate injections of the four essential oils. RI, retention index denotes reference retention indices obtained from established databases and published literature for stationary phases comparable to the Rxi-5Sil MS column. These values were used only as supporting evidence to assess the plausibility of the library-based assignments and were not experimentally determined in the present study. ID, Identification confidence: A, high EI-MS agreement with plausible retention behavior; B, tentative isomer/common-name assignment or moderate evidence; C, unidentified because of low similarity and/or retention conflict.
