# Supplementary material for: Comparison of Chemical Compositions and Antioxidant Activity of Essential Oils from Litsea Cubeba, Cinnamon, Anise, and Eucalyptus
Source: Molecules. 2023 Jun 28;28(13):5051. doi: 10.3390/molecules28135051 (PMC10343171; doi:10.3390/molecules28135051)
Supplement: Supplementary file 1 [file molecules-28-05051-s001.zip › molecules-2454450-supplementary.pdf]

Supplementary Table S1. Peak-level NIST11 library matching information for the four original GC-MS reports.

S1 No.	Table 1 No.	Oil/peak	RT (min)	Area (%)	Final assignment	Assigned CAS	Selected NIST11 hit	NIST CAS	Hit/SI	RI_NIST	Basis
1	2	LCO/1	6.710	1.46	α-Pinene	80-56-8	α-Pinene	80-56-8	1/97	948	Tentative
2	3	LCO/2	7.251	0.35	Camphene	79-92-5	Camphene	79-92-5	1/96	943	Direct
3	5	LCO/3	8.075	1.08	Sabinene	3387-41-5	Bicyclo[3.1.0]hexane, 4-methylene-1-(1-methylethyl)-	3387-41-5	1/95	897	Direct
4	6	LCO/4	8.194	1.13	β-Pinene	127-91-3	β-Pinene	127-91-3	1/97	943	Direct
5	7	LCO/5	8.570	1.80	6-Methyl-5-hepten-2-one	110-93-0	5-Hepten-2-one, 6-methyl-	110-93-0	1/96	938	Direct
6	8	LCO/6	8.695	0.71	β-Myrcene	123-35-3	β-Myrcene	123-35-3	1/94	958	Direct
7	13	LCO/7	9.950	12.39	Limonene	138-86-3	D-Limonene	5989-27-5	1/95	1018	Achiral assignment
8	16	LCO/8	12.229	2.08	Linalool	78-70-6	1,6-Octadien-3-ol, 3,7-dimethyl-	78-70-6	1/97	1082	Direct
9	17	LCO/9	13.294	0.26	Unidentified LCO-9 (best MS match: 3,3,5-trimethyl-1,4-hexadiene)	-	1,4-Hexadiene, 3,3,5-trimethyl-	74753-00-7	1/91	807	Candidate only
10	19	LCO/10	13.535	2.47	Citronellal	106-23-0	6-Octenal, 3,7-dimethyl-, (R)-	2385-77-5	1/98	1125	Achiral assignment
11	21	LCO/11	13.791	1.32	2,4,6-Trimethylcyclohex-3-ene-1-carbaldehyde (tentative)	1423-46-7	3-Cyclohexene-1-carboxaldehyde, 2,4,6-trimethyl-	1423-46-7	1/88	1163	Tentative
12	23	LCO/12	14.201	0.10	endo-Borneol	507-70-0	endo-Borneol	507-70-0	1/94	1138	Direct
13	24	LCO/13	14.292	2.62	Unresolved oxygenated monoterpene LCO-13 (best MS match: trans-4,5-epoxycarane)	-	Carane, 4,5-epoxy-, trans	6909-20-2	1/88	948	Candidate only
14	27	LCO/14	14.767	0.78	α-Terpineol	10482-56-1	L-α-Terpineol	10482-56-1	1/95	1143	Direct
15	29	LCO/15	15.636	0.72	Nerol	106-25-2	2,6-Octadien-1-ol, 3,7-dimethyl-, (Z)-	106-25-2	1/95	1228	Direct
16	30	LCO/16	15.891	27.97	Neral (β-citral)	106-26-3	2,6-Octadienal, 3,7-dimethyl-, (Z)-	106-26-3	1/98	1174	Direct
17	32	LCO/17	16.180	0.13	Piperitone	89-81-6	2-Cyclohexen-1-one, 3-methyl-6-(1-methylethyl)-	89-81-6	1/93	1158	Direct
18	34	LCO/18	16.322	1.93	Geraniol	106-24-1	Geraniol	106-24-1	1/95	1228	Direct
19	35	LCO/19	16.659	35.58	Geranial (α-citral)	141-27-5	2,6-Octadienal, 3,7-dimethyl-, (E)-	141-27-5	1/97	1174	Direct

S1 No.	Table 1 No.	Oil/peak	RT (min)	Area (%)	Final assignment	Assigned CAS	Selected NIST11 hit	NIST CAS	Hit/SI	RI_NIST	Basis
20	37	LCO/20	17.086	0.32	Unidentified LCO-20 (best MS match: limonene oxide)	-	7-Oxabicyclo[4.1.0]heptane, 1-methyl-4-(1-methylethenyl)-	1195-92-2	1/85	1031	Candidate only
21	39	LCO/21	18.101	0.24	Unidentified LCO-21 (best MS match: 2,7-dimethyl-2,7-octanediol; literature-reported candidate)	-	2,7-Dimethyl-2,7-octanediol	19781-07-8	1/84	1199	Candidate only
22	40	LCO/22	18.704	0.21	Unidentified LCO-22 (best MS match: 2,4,4,7-tetramethyl-5,7-octadien-3-ol)	-	5,7-Octadien-3-ol, 2,4,4,7-tetramethyl-, (E)-	77142-78-0	1/81	1203	Candidate only
23	41	LCO/23	18.920	0.39	Unidentified LCO-23 (best MS match: 6-hepten-3-ol)	-	6-Hepten-3-ol	19781-77-2	1/82	869	Candidate only
24	44	LCO/24	19.284	0.33	β-Elemene	515-13-9	β-Elemene	515-13-9	1/96	1398	Direct
25	46	LCO/25	19.938	2.37	β-Caryophyllene	87-44-5	Caryophyllene	87-44-5	1/96	1494	Direct
26	60	LCO/26	23.301	0.56	Caryophyllene oxide	1139-30-6	Caryophyllene oxide	1139-30-6	1/94	1507	Direct
27	64	LCO/27	31.513	0.10	trans-Geranylgeraniol	24034-73-9	trans-Geranylgeraniol	24034-73-9	1/88	2192	Tentative
28	65	LCO/28	31.632	0.20	Unidentified LCO-28 (best MS match: isoaromadendrene epoxide)	-	Isoaromadendrene epoxide	-	1/84	1281	Candidate only
29	66	LCO/29	32.762	0.22	Unidentified LCO-29 (best MS match: neric acid)	-	Neric acid	4613-38-1	1/83	1342	Candidate only
30	67	LCO/30	32.892	0.16	Unidentified LCO-30	-	2-Buten-1-one, 1-(2,2,5a-trimethylperhydro-1-benzoxiren-1-yl)	-	1/81	1454	Candidate only
31	4	CO/1	7.750	1.06	Benzaldehyde	100-52-7	Benzaldehyde	100-52-7	1/97	982	Direct
32	22	CO/2	13.793	0.74	3-Phenylpropanal (hydrocinnamaldehyde)	104-53-0	Benzenepropanal	104-53-0	1/98	1181	Direct
33	28	CO/3	15.290	0.39	(Z)-Cinnamaldehyde (cis; tentative)	57194-69-1	Cinnamaldehyde, (E)-	14371-10-9	1/95	1189	Isomer/RI
34	36	CO/4	16.884	84.86	(E)-Cinnamaldehyde (trans)	14371-10-9	Cinnamaldehyde, (E)-	14371-10-9	2/97	1189	Direct
35	42	CO/5	18.961	0.57	α-Copaene	3856-25-5	Copaene	3856-25-5	3/93	1221	Tentative isomer
36	46	CO/6	19.925	0.18	(E)-Caryophyllene (tentative)	87-44-5	Caryophyllene	87-44-5	3/94	1494	Alt. hit/RT
37	47	CO/7	20.221	0.12	trans-α-Bergamotene	13474-59-4	trans-α-Bergamotene	13474-59-4	1/95	1430	Isomer/RI
38	48	CO/8	20.380	6.43	Coumarin (tentative)	91-64-5	Coumarin	91-64-5	2/96	1374	Alt. hit/RI
39	49	CO/9	20.522	1.46	Cinnamyl acetate	103-54-8	Acetic acid, cinnamyl ester	103-54-8	1/96	1367	Direct

S1 No.	Table 1 No.	Oil/peak	RT (min)	Area (%)	Final assignment	Assigned CAS	Selected NIST11 hit	NIST CAS	Hit/SI	RI_NIST	Basis
40	50	CO/10	20.793	0.19	Alloaromadendrene	25246-27-9	Alloaromadendrene	25246-27-9	1/94	1386	Direct
41	51	CO/11	21.111	0.22	γ-Muurolene	30021-74-0	γ-Muurolene	30021-74-0	1/93	1435	Direct
42	53	CO/12	21.608	0.12	α-Amorphene	483-75-0	Naphthalene, 1,2,4a,5,6,8a-hexahydro-4,7-dimethyl-1-(1-methylethyl)-	483-75-0	1/93	1440	Tentative isomer
43	54	CO/13	21.797	0.38	β-Bisabolene	495-61-4	β-Bisabolene	495-61-4	1/96	1500	Direct
44	55	CO/14	21.904	0.17	Unidentified (candidate: β-copaene)	-	β-copaene	-	1/91	1216	Candidate only
45	56	CO/15	22.011	0.35	δ-Cadinene	483-76-1	δ-Cadinene	483-76-1	1/90	1469	Direct
46	57	CO/16	22.183	0.16	Unidentified CO-16 (best MS match: 4,8,11,11-tetramethyltricyclo[7.2.0.0(3,8)]undec-4-ene)	-	Tricyclo[7.2.0.0(3,8)]undec-4-ene, 4,8,11,11-tetramethyl-	-	1/85	1403	Candidate only
47	58	CO/17	22.267	1.76	2-Methoxycinnamaldehyde (tentative)	1504-74-1	2-Propenal, 3-(2-methoxyphenyl)-	1504-74-1	1/97	1378	MS + RI review
48	59	CO/18	22.924	0.34	(E)-Nerolidol	40716-66-3	1,6,10-Dodecatrien-3-ol, 3,7,11-trimethyl-, (E)-	40716-66-3	1/95	1564	Direct
49	60	CO/19	23.297	0.35	Caryophyllene oxide	1139-30-6	Caryophyllene oxide	1139-30-6	1/92	1507	Direct
50	63	CO/20	30.762	0.15	Unidentified CO-20 (best MS match: β-selinene; retention conflict)	-	β-Selinene	17066-67-0	1/87	1469	Candidate only
51	2	AO/1	6.704	0.98	α-Pinene	80-56-8	α-Pinene	80-56-8	1/97	948	Tentative
52	9	AO/2	9.162	0.48	α-Phellandrene	99-83-2	α-Phellandrene	99-83-2	1/96	969	Direct
53	10	AO/3	9.236	0.37	3-Carene (tentative)	13466-78-9	3-Carene	13466-78-9	4/95	948	Alt. hit/RT
54	13	AO/4	9.908	2.20	Limonene	138-86-3	D-Limonene	5989-27-5	1/93	1018	Achiral assignment
55	14	AO/5	9.967	0.70	1,8-Cineole (eucalyptol)	470-82-6	Eucalyptol	470-82-6	1/86	1059	Direct
56	16	AO/6	12.214	2.21	Linalool	78-70-6	1,6-Octadien-3-ol, 3,7-dimethyl-	78-70-6	1/97	1082	Direct
57	25	AO/7	14.303	0.30	Terpinen-4-ol	562-74-3	Terpinen-4-ol	562-74-3	1/96	1137	Direct
58	26	AO/8	14.746	6.15	Estragole	140-67-0	Estragole	140-67-0	1/96	1172	Direct
59	31	AO/9	16.123	0.45	(Z)-Anethole (cis; tentative)	25679-28-1	Anethole	104-46-1	3/94	1190	Alt. hit/RI
60	33	AO/10	16.256	1.06	p-Anisaldehyde	123-11-5	Benzaldehyde, 4-methoxy-	123-11-5	1/97	1171	Direct

S1 No.	Table 1 No.	Oil/peak	RT (min)	Area (%)	Final assignment	Assigned CAS	Selected NIST11 hit	NIST CAS	Hit/SI	RI_NIST	Basis
61	38	AO/11	17.160	78.51	(E)-Anethole (trans)	4180-23-8	Anethole	104-46-1	1/98	1190	Stereochemistry
62	43	AO/12	19.094	0.32	Unidentified AO-12 (best MS match: 2-phenyloxiranemethanol)	-	Oxiranemethanol, 2-phenyl-	141248-89-7	1/78	1300	Candidate only
63	45	AO/13	19.794	0.18	cis-α-Bergamotene (tentative)	18252-46-5	Bicyclo[3.1.1]hept-2-ene, 2,6-dimethyl-6-(4-methyl-3-pentenyl)-	17699-05-7	1/94	1430	Isomer/RI
64	46	AO/14	19.927	0.80	β-Caryophyllene	87-44-5	Caryophyllene	87-44-5	1/95	1494	Direct
65	47	AO/15	20.223	0.92	trans-α-Bergamotene	13474-59-4	trans-α-Bergamotene	13474-59-4	2/94	1430	Isomer/RI
66	52	AO/16	21.536	0.24	Unidentified (candidate: Germacrene B)	-	1,5-Cyclodecadiene, 1,5-dimethyl-8-(1-methylethylidene)-, (E,E)-	15423-57-1	1/91	1603	Candidate only
67	54	AO/17	21.796	0.20	β-Bisabolene	495-61-4	β-Bisabolene	495-61-4	1/96	1500	Direct
68	59	AO/18	22.920	0.24	(E)-Nerolidol	40716-66-3	1,6,10-Dodecatrien-3-ol, 3,7,11-trimethyl-, (E)-	40716-66-3	1/95	1564	Direct
69	61	AO/19	24.757	0.23	α-Cadinol	481-34-5	α-Cadinol	481-34-5	1/87	1580	Tentative
70	62	AO/20	25.133	3.47	Foeniculin	78259-41-3	1-(3-Methyl-2-butenoxy)-4-(1-propenyl)benzene	78259-41-3	1/91	1572	Direct
71	1	EUC/1	2.015	0.03	Unidentified EUC-1 (best MS match: 1-methylhexyl hydroperoxide)	-	Hydroperoxide, 1-methylhexyl	762-46-9	1/79	1013	Candidate only
72	2	EUC/2	6.707	0.30	α-Pinene	80-56-8	α-Pinene	80-56-8	2/96	948	Isomer/RT
73	3	EUC/3	7.251	0.16	Camphene	79-92-5	Camphene	79-92-5	1/95	943	Direct
74	5	EUC/4	8.072	2.19	Sabinene	3387-41-5	Bicyclo[3.1.0]hexane, 4-methylene-1-(1-methylethyl)-	3387-41-5	1/94	897	Direct
75	6	EUC/5	8.190	1.34	β-Pinene	127-91-3	β-Pinene	127-91-3	1/97	943	Direct
76	8	EUC/6	8.700	4.58	β-Myrcene	123-35-3	β-Myrcene	123-35-3	1/96	958	Direct
77	9	EUC/7	9.171	1.97	α-Phellandrene	99-83-2	α-Phellandrene	99-83-2	1/96	969	Direct
78	11	EUC/8	9.530	5.35	4-Carene (tentative)	-	(+)-4-Carene	29050-33-7	1/96	919	Achiral/tentative
79	12	EUC/9	9.825	1.81	Cymene isomer (tentative)	-	Benzene, 1-methyl-3-(1-methylethyl)-	535-77-3	1/95	1042	Isomer unresolved
80	14	EUC/10	10.092	81.79	1,8-Cineole (eucalyptol)	470-82-6	Eucalyptol	470-82-6	1/93	1059	Direct

S1 No.	Table 1 No.	Oil/peak	RT (min)	Area (%)	Final assignment	Assigned CAS	Selected NIST11 hit	NIST CAS	Hit/SI	RI_NIST	Basis
81	15	EUC/11	10.818	0.19	γ-Terpinene	99-85-4	γ-Terpinene	99-85-4	1/96	998	Direct
82	18	EUC/12	13.330	0.25	Unidentified EUC-12 (best MS match: 2,3,5-trimethyl-1,3-hexadiene)	-	1,3-Hexadiene, 2,3,5-trimethyl-	61142-34-5	1/85	804	Candidate only
83	20	EUC/13	13.541	0.05	Unidentified EUC-13 (best MS match: 3,3,5-trimethylcyclohexyl methacrylate)	-	2-Propenoic acid, 2-methyl-, 3,3,5-trimethylcyclohexyl ester	7779-31-9	1/78	1409	Candidate only

Note:

Each row represents one peak in the four supplied PDF reports. Table 1 No. links the original peak to the consolidated main-table row. Hit/SI gives the selected NIST11 hit rank and similarity index.

RI_NIST and NIST CAS are copied from the selected library hit. RI_NIST is a library record, not a same-run n-alkane-calibrated RI. A lower-ranked hit was selected only when it had comparable spectral similarity and the retention/elution evidence was materially stronger.

For ID C peaks, Assigned CAS is a hyphen and the NIST candidate is traceability evidence only.

Basis codes: Direct, direct library support; Achiral assignment, stereochemistry removed because the column is non-chiral; Alt. hit/RT, tied or near-tied alternative supported by elution order; Isomer unresolved, positional isomer not distinguished; Candidate only, unresolved peak.

Supplementary Table S2. Row-level provenance of the RI values reported in Table 1 and the corresponding identification assessments.

Row link	Table 1 entry	Literature/database source evidence (unaltered)			Reporting information for the revised Table 1	
Table 1 No.	Compound / ID	Original reported value(s)	Source ID and exact location	Stationary phase / RI record	RI reported in Table 1	Reporting basis / identification assessment
1	Unidentified EUC-1 (best MS match: 1-methylhexyl hydroperoxide) [ID C]	— (no source value adopted)	—	—	n.a.	No literature/database RI was adopted because the peak remains unidentified (ID C); a library candidate or RI_NIST in S1 is traceability evidence only, not an assigned compound RI.
2	α-Pinene [ID A; EUC B]	N=765; mean=936.1; SD=8.0; median=937; 50% RI=933–939; 90% RI=921–944	B2011-T4; PDF p. 27 (article p. 043101-27)	Dimethylsilicone stationary phase with 5% phenyl groups; published statistical compilation	936	Published mean 936.1 rounded to 936 (nearest integer; 0.5 upward). The compound assignment remains tentative (ID B).
3	Camphene [ID A]	N=480; mean=950.3; SD=7.0; median=952; 50% RI=948–954; 90% RI=936–959	B2011-T4; PDF p. 27 (article p. 043101-27)	Dimethylsilicone stationary phase with 5% phenyl groups; published statistical compilation	950	Published mean 950.3 rounded to 950 (nearest integer; 0.5 upward).
4	Benzaldehyde [ID A]	N=304; mean=962.7; SD=10.4; median=962; 50% RI=959–967; 90% RI=947–982	B2011-T4; PDF p. 27 (article p. 043101-27)	Dimethylsilicone stationary phase with 5% phenyl groups; published statistical compilation	963	Published mean 962.7 rounded to 963 (nearest integer; 0.5 upward).
5	Sabinene [ID A]	N=472; mean=973.0; SD=7.0; median=975; 50% RI=971–976; 90% RI=961–981	B2011-T4; PDF p. 27 (article p. 043101-27)	Dimethylsilicone stationary phase with 5% phenyl groups; published statistical compilation	973	Published mean 973.0 rounded to 973 (nearest integer; 0.5 upward).
6	β-Pinene [ID A]	N=643; mean=977.7; SD=7.1; median=979; 50% RI=975–980; 90% RI=964–988	B2011-T4; PDF p. 27 (article p. 043101-27)	Dimethylsilicone stationary phase with 5% phenyl groups; published statistical compilation	978	Published mean 977.7 rounded to 978 (nearest integer; 0.5 upward).
7	6-Methyl-5-hepten-2-one [ID A]	N=163; mean=985.9; SD=5.7; median=986; 50% RI=984–988; 90% RI=977–995	B2011-T4; PDF p. 27 (article p. 043101-27)	Dimethylsilicone stationary phase with 5% phenyl groups; published statistical compilation	986	Published mean 985.9 rounded to 986 (nearest integer; 0.5 upward).
8	β-Myrcene [ID A]	N=655; mean=989.2; SD=6.2; median=991; 50% RI=988–992; 90% RI=980–995	B2011-T4; PDF p. 27 (article p. 043101-27)	Dimethylsilicone stationary phase with 5% phenyl groups; published statistical compilation	989	Published mean 989.2 rounded to 989 (nearest integer; 0.5 upward).

Row link	Table 1 entry	Literature/database source evidence (unaltered)			Reporting information for the revised Table 1	
Table 1 No.	Compound / ID	Original reported value(s)	Source ID and exact location	Stationary phase / RI record	RI reported in Table 1	Reporting basis / identification assessment
9	α-Phellandrene [ID A]	N=395; mean=1004.1; SD=6.8; median=1004; 50% RI=1002–1006; 90% RI=995–1013	B2011-T4; PDF p. 27 (article p. 043101-27)	Dimethylsilicone stationary phase with 5% phenyl groups; published statistical compilation	1004	Published mean 1004.1 rounded to 1004 (nearest integer; 0.5 upward).
10	3-Carene (tentative) [ID B]	B2011 Table 4: N=267; mean=1011.3; SD=6.4; median=1011; 50% RI=1008–1013; 90% RI=1002–1025.	B2011-T4; PDF p. 27.	Dimethylsilicone stationary phase with 5% phenyl groups; published statistical compilation.	1011	The tied fourth NIST hit (SI 95) was selected because 3-carene fits the observed position before limonene. trans-β-Ocimene (B2011 mean 1047.8) is incompatible with this early RT. Assignment remains tentative (ID B).
11	4-Carene (tentative) [ID B]	LIU2013 Table 1: RI=1018.	LIU2013-T1; article Table 1.	HP-5MS, 30 m × 0.25 mm × 0.25 μm; RI determined against C8–C24 n-alkanes under the same GC conditions.	1018	The published HP-5MS value was adopted. The non-chiral Rxi-5Sil MS run cannot establish the (+) enantiomer, and the library also returned 2-carene; the entry is therefore reported as generic 4-carene and ID B.
12	Cymene isomer (tentative; m-/p-cymene unresolved) [ID B]	Present PDF/NIST11: m-cymene and p-cymene both SI=95; o-cymene SI=94.	Original EUC PDF, peak 9 (RT 9.825 min).	Rxi-5Sil MS EI-MS library candidates; no same-run n-alkane RI and no authentic standard.	n.a.	The positional isomer cannot be resolved by the reported evidence. A single-isomer RI was not adopted; the entry is reported as Cymene isomer (tentative), ID B.
13	Limonene [ID A]	N=753; mean=1029.5; SD=6.9; median=1030; 50% RI=1027–1032; 90% RI=1019–1039	B2011-T4; PDF p. 28 (article p. 043101-28)	Dimethylsilicone stationary phase with 5% phenyl groups; published statistical compilation	1030	The B2011 mean was rounded to 1030. The achiral column and EI-MS cannot establish D/(R) stereochemistry, so the name is reported as Limonene.
14	1,8-Cineole (eucalyptol) [ID A]	N=444; mean=1031.8; SD=6.9; median=1032; 50% RI=1030–1034; 90% RI=1021–1044	B2011-T4; PDF p. 28 (article p. 043101-28)	Dimethylsilicone stationary phase with 5% phenyl groups; published statistical compilation	1032	Published mean 1031.8 rounded to 1032 (nearest integer; 0.5 upward).
15	γ-Terpinene [ID A]	N=575; mean=1059.7; SD=7.5; median=1060; 50% RI=1057–1062; 90% RI=1049–1069	B2011-T4; PDF p. 28 (article p. 043101-28)	Dimethylsilicone stationary phase with 5% phenyl groups; published statistical compilation	1060	Published mean 1059.7 rounded to 1060 (nearest integer; 0.5 upward).
16	Linalool [ID A]	N=716; mean=1099.0; SD=6.8; median=1099; 50% RI=1097–1102; 90% RI=1088–1109	B2011-T4; PDF p. 28 (article p. 043101-28)	Dimethylsilicone stationary phase with 5% phenyl groups; published statistical compilation	1099	Published mean 1099.0 rounded to 1099 (nearest integer; 0.5 upward).

Row link	Table 1 entry	Literature/database source evidence (unaltered)			Reporting information for the revised Table 1	
Table 1 No.	Compound / ID	Original reported value(s)	Source ID and exact location	Stationary phase / RI record	RI reported in Table 1	Reporting basis / identification assessment
17	Unidentified LCO-9 (best MS match: 3,3,5-trimethyl-1,4-hexadiene) [ID C]	— (no source value adopted)	—	—	n.a.	No literature/database RI was adopted because the peak remains unidentified (ID C); a library candidate or RI_NIST in S1 is traceability evidence only, not an assigned compound RI.
18	Unidentified EUC-12 (best MS match: 2,3,5-trimethyl-1,3-hexadiene) [ID C]	— (no source value adopted)	—	—	n.a.	No literature/database RI was adopted because the peak remains unidentified (ID C); a library candidate or RI_NIST in S1 is traceability evidence only, not an assigned compound RI.
19	Citronellal [ID A]	N=77; mean=1153.7; SD=5.8; median=1153; 50% RI=1152–1156; 90% RI=1144–1162	B2011-T4; PDF p. 29 (article p. 043101-29)	Dimethylsilicone stationary phase with 5% phenyl groups; published statistical compilation	1154	The B2011 mean was rounded to 1154. The achiral column and EI-MS cannot establish the (R)-(+)-enantiomer, so the name is reported as Citronellal.
20	Unidentified EUC-13 (best MS match: 3,3,5-trimethylcyclohexyl methacrylate) [ID C]	— (no source value adopted)	—	—	n.a.	No literature/database RI was adopted because the peak remains unidentified (ID C); a library candidate or RI_NIST in S1 is traceability evidence only, not an assigned compound RI.
21	2,4,6-Trimethylcyclohex-3-ene-1-carbaldehyde (tentative) [ID B]	— (no source value adopted)	—	—	n.a.	No directly traceable comparable 5%-phenyl low-polarity source value was verified in this audit; Table 1 therefore remains n.a. This is an evidence-availability decision, not an estimated RI.
22	3-Phenylpropanal (hydrocinnamaldehyde) [ID A]	ORO2020: RI_exp=1163; RI_lit=1163.	ORO2020, Table 6/7.	HP-5ms, 30 m × 0.25 mm × 0.25 µm, 5% diphenyl/95% dimethylpolysiloxane; He 0.9 mL/min; 60–246 °C at 3 °C/min; C8–C34 n-alkanes run under the same conditions.	1163	The experimentally measured, method-comparable HP-5ms value was adopted without alteration.
23	endo-Borneol [ID A]	N=322; mean=1166.2; SD=7.9; median=1166; 50% RI=1164–1169; 90% RI=1152–1177	B2011-T4; PDF p. 29 (article p. 043101-29)	Dimethylsilicone stationary phase with 5% phenyl groups; published statistical compilation	1166	Published mean 1166.2 rounded to 1166 (nearest integer; 0.5 upward).
24	Unresolved oxygenated monoterpene LCO-13 (best MS match: trans-4,5-epoxycarane) [ID C]	— (no source value adopted)	—	—	n.a.	No literature/database RI was adopted because the peak remains unidentified (ID C); a library candidate or RI_NIST in S1 is traceability evidence only, not an assigned compound RI.

Row link	Table 1 entry	Literature/database source evidence (unaltered)			Reporting information for the revised Table 1	
Table 1 No.	Compound / ID	Original reported value(s)	Source ID and exact location	Stationary phase / RI record	RI reported in Table 1	Reporting basis / identification assessment
25	Terpinen-4-ol [ID A]	N=593; mean=1177.1; SD=9.0; median=1177; 50% RI=1175–1179; 90% RI=1165–1189	B2011-T4; PDF p. 29 (article p. 043101-29)	Dimethylsilicone stationary phase with 5% phenyl groups; published statistical compilation	1177	Published mean 1177.1 rounded to 1177 (nearest integer; 0.5 upward).
26	Estragole [ID A]	N=69; mean=1195.8; SD=5.2; median=1196; 50% RI=1195–1198; 90% RI=1184–1204	B2011-T4; PDF p. 30 (article p. 043101-30)	Dimethylsilicone stationary phase with 5% phenyl groups; published statistical compilation	1196	Published mean 1195.8 rounded to 1196 (nearest integer; 0.5 upward).
27	α-Terpineol [ID A]	N=624; mean=1189.7; SD=9.4; median=1189; 50% RI=1188–1192; 90% RI=1178–1203	B2011-T4; PDF p. 30 (article p. 043101-30)	Dimethylsilicone stationary phase with 5% phenyl groups; published statistical compilation	1190	Published mean 1189.7 rounded to 1190 (nearest integer; 0.5 upward).
28	(Z)-Cinnamaldehyde (cis; tentative) [ID B]	N=6; mean=1215.2; SD=7.6; 90% RI=1200–1219; median and 50% RI not reported	B2011-T4; PDF p. 30 (article p. 043101-30)	Dimethylsilicone stationary phase with 5% phenyl groups; published statistical compilation	1215	Published mean 1215.2 rounded to 1215 (nearest integer; 0.5 upward). The compound assignment remains tentative (ID B).
29	Nerol [ID A]	N=125; mean=1228.9; SD=9.5; median=1228; 50% RI=1226–1230; 90% RI=1216–1250	B2011-T4; PDF p. 30 (article p. 043101-30)	Dimethylsilicone stationary phase with 5% phenyl groups; published statistical compilation	1229	Published mean 1228.9 rounded to 1229 (nearest integer; 0.5 upward).
30	Neral (β-citral) [ID A]	N=116; mean=1242.1; SD=9.6; median=1240; 50% RI=1237–1244; 90% RI=1231–1269	B2011-T4; PDF p. 30 (article p. 043101-30)	Dimethylsilicone stationary phase with 5% phenyl groups; published statistical compilation	1242	Published mean 1242.1 rounded to 1242 (nearest integer; 0.5 upward).
31	(Z)-Anethole (cis; tentative) [ID B]	BOS2024: RI_exp=1251; RI_lit=1249. MUR2024: KI=1249.	BOS2024-T2, PDF p. 7 (printed p. 133); MUR2024-T1, PDF p. 3	BOS2024: 30 m DB-5, C8-C40 n-alkanes for RI_exp; RI_lit from Adams/EssentialOil databases. MUR2024: Adams linear DB-5 KI	1249	The literature/reference value 1249 was used; BOS2024's sample-specific RI_exp 1251 was not substituted into Table 1. No rounding. Assignment remains tentative (ID B).
32	Piperitone [ID A]	N=91; mean=1253.6; SD=6.7; median=1252; 50% RI=1251–1255; 90% RI=1245–1266	B2011-T4; PDF p. 30 (article p. 043101-30)	Dimethylsilicone stationary phase with 5% phenyl groups; published statistical compilation	1254	Published mean 1253.6 rounded to 1254 (nearest integer; 0.5 upward).

Row link	Table 1 entry	Literature/database source evidence (unaltered)			Reporting information for the revised Table 1	
Table 1 No.	Compound / ID	Original reported value(s)	Source ID and exact location	Stationary phase / RI record	RI reported in Table 1	Reporting basis / identification assessment
33	p-Anisaldehyde [ID A]	N=35; mean=1251.8; SD=8.9; median=1251; 50% RI=1247–1255; 90% RI=1239–1266	B2011-T4; PDF p. 30 (article p. 043101-30)	Dimethylsilicone stationary phase with 5% phenyl groups; published statistical compilation	1252	Published mean 1251.8 rounded to 1252 (nearest integer; 0.5 upward).
34	Geraniol [ID A]	N=247; mean=1254.9; SD=9.0; median=1255; 50% RI=1253–1258; 90% RI=1238–1269	B2011-T4; PDF p. 30 (article p. 043101-30)	Dimethylsilicone stationary phase with 5% phenyl groups; published statistical compilation	1255	Published mean 1254.9 rounded to 1255 (nearest integer; 0.5 upward).
35	Geranial (α-citral) [ID A]	N=142; mean=1270.3; SD=9.8; median=1270; 50% RI=1267–1273; 90% RI=1252–1291	B2011-T4; PDF p. 30 (article p. 043101-30)	Dimethylsilicone stationary phase with 5% phenyl groups; published statistical compilation	1270	Published mean 1270.3 rounded to 1270 (nearest integer; 0.5 upward).
36	(E)-Cinnamaldehyde (trans) [ID A]	N=20; mean=1271.3; SD=8.1; median=1270; 50% RI=1267–1274; 90% RI=1264–1287	B2011-T4; PDF p. 30 (article p. 043101-30)	Dimethylsilicone stationary phase with 5% phenyl groups; published statistical compilation	1271	Published mean 1271.3 rounded to 1271 (nearest integer; 0.5 upward).
37	Unidentified LCO-20 (best MS match: limonene oxide) [ID C]	cis-limonene oxide: N=41; mean=1134.0; SD=6.2; median=1134; 50% RI=1131–1138; 90% RI=1122–1144. trans-limonene oxide: N=32; mean=1137.5; SD=6.9; median=1138; 50% RI=1134–1142; 90% RI=1126–1149.	B2011-T4; PDF p. 29 (article p. 043101-29)	Dimethylsilicone stationary phase with 5% phenyl groups; published statistical compilation	n.a.	Candidate-only source check. The peak remains unidentified (ID C); no candidate RI is displayed in Table 1. The former constructed 1134-1138 dagger entry was removed; no new mean or range was created.
38	(E)-Anethole (trans) [ID B]	N=50; mean=1285.2; SD=8.0; median=1284; 50% RI=1283–1287; 90% RI=1273–1303	B2011-T4; PDF p. 31 (article p. 043101-31)	Dimethylsilicone stationary phase with 5% phenyl groups; published statistical compilation	1285	Published mean 1285.2 rounded to 1285 (nearest integer; 0.5 upward). The compound assignment remains tentative (ID B).
39	Unidentified LCO-21 (best MS match: 2,7-dimethyl-2,7-octanediol; literature-reported candidate) [ID C]	— (no source value adopted)	—	—	n.a.	No literature/database RI was adopted because the peak remains unidentified (ID C); a library candidate or RI_NIST in S1 is traceability evidence only, not an assigned compound RI.
40	Unidentified LCO-22 (best MS match: 2,4,4,7-tetramethyl-5,7-octadien-3-ol) [ID C]	— (no source value adopted)	—	—	n.a.	No literature/database RI was adopted because the peak remains unidentified (ID C); a library candidate or RI_NIST in S1 is traceability evidence only, not an assigned compound RI.

Row link	Table 1 entry	Literature/database source evidence (unaltered)			Reporting information for the revised Table 1	
Table 1 No.	Compound / ID	Original reported value(s)	Source ID and exact location	Stationary phase / RI record	RI reported in Table 1	Reporting basis / identification assessment
41	Unidentified LCO-23 (best MS match: 6-hepten-3-ol) [ID C]	— (no source value adopted)	—	—	n.a.	No literature/database RI was adopted because the peak remains unidentified (ID C); a library candidate or RI_NIST in S1 is traceability evidence only, not an assigned compound RI.
42	α-Copaene [ID B]	N=524; mean=1376.2; SD=9.2; median=1376; 50% RI=1374–1378; 90% RI=1363–1391	B2011-T4; PDF p. 31 (article p. 043101-31)	Dimethylsilicone stationary phase with 5% phenyl groups; published statistical compilation	1376	The B2011 mean was rounded to 1376. Because the original peak gave tied/near-tied copaene and cubebene candidates and the selected hit was not rank 1, confidence was downgraded to ID B.
43	Unidentified AO-12 (best MS match: 2-phenyloxiranemethanol) [ID C]	— (no source value adopted)	—	—	n.a.	No literature/database RI was adopted because the peak remains unidentified (ID C); a library candidate or RI_NIST in S1 is traceability evidence only, not an assigned compound RI.
44	β-Elemene [ID A]	N=368; mean=1390.4; SD=9.0; median=1391; 50% RI=1388–1393; 90% RI=1374–1402	B2011-T4; PDF p. 31 (article p. 043101-31)	Dimethylsilicone stationary phase with 5% phenyl groups; published statistical compilation	1390	Published mean 1390.4 rounded to 1390 (nearest integer; 0.5 upward).
45	cis-α-Bergamotene (tentative) [ID B]	N=50; mean=1414.5; SD=8.7; median=1415; 50% RI=1409–1416; 90% RI=1403–1433	B2011-T4; PDF p. 32 (article p. 043101-32)	Dimethylsilicone stationary phase with 5% phenyl groups; published statistical compilation	1415	Published mean 1414.5 rounded to 1415 (nearest integer; 0.5 upward). The compound assignment remains tentative (ID B).
46	(E)-Caryophyllene (β-caryophyllene) [ID A; CO B]	N=741; mean=1420.1; SD=10.4; median=1419; 50% RI=1417–1422; 90% RI=1405–1440 ORO2020: (E)-caryophyllene RI_exp=1413; RI_lit=1417.	B2011-T4; PDF p. 32 (article p. 043101-32) ORO2020, Tables 6–7.	B2011: 5%-phenyl dimethylsilicone statistical compilation. ORO2020: HP-5ms, 30 m × 0.25 mm × 0.25 μm, n-alkane RI.	1420	B2011 mean was rounded to 1420. The CO peak at RT 19.925 min coelutes with the LCO/AO (E)-caryophyllene peaks at 19.938 and 19.927 min; its library E/Z hits were nearly tied. The CO area was consolidated into this row but remains ID B; LCO/AO remain A.
47	trans-α-Bergamotene [ID B]	N=141; mean=1434.5; SD=8.4; median=1436; 50% RI=1432–1438; 90% RI=1416–1444	B2011-T4; PDF p. 32 (article p. 043101-32)	Dimethylsilicone stationary phase with 5% phenyl groups; published statistical compilation	1435	Published mean 1434.5 rounded to 1435 (nearest integer; 0.5 upward). The compound assignment remains tentative (ID B).
48	Coumarin (tentative) [ID B]	ORO2020: coumarin RI_exp=1456; RI_lit=1432. NIST-SRD69 comparable HP-5MS record: I=1432.	ORO2020, Table 6/7; NIST-SRD69 C91645.	ORO2020 HP-5ms with same-run C8–C34 n-alkanes; NIST HP-5MS temperature-ramp record.	1432	The traceable literature/reference value 1432 was retained, while the external study's sample-specific RI_exp=1456 is shown separately. Assignment remains tentative (ID B).

Row link	Table 1 entry	Literature/database source evidence (unaltered)			Reporting information for the revised Table 1	
**Table 1 No.**	**Compound / ID**	**Original reported value(s)**	**Source ID and exact location**	**Stationary phase / RI record**	**RI reported in Table 1**	**Reporting basis / identification assessment**
49	Cinnamyl acetate [ID A]	ORO2020: (E)-cinnamyl acetate RI_exp=1448; RI_lit=1443.	ORO2020, Tables 6–7.	HP-5ms, 30 m × 0.25 mm × 0.25 μm; C8–C34 n-alkanes run under the same conditions.	1448	The method-comparable HP-5ms RI_exp value of 1448 from the cited publication was selected as the RI reported in Table 1; the previously selected NIST value of 1446 remains available in the audit trail but was not used for Table 1.
50	Alloaromadendrene [ID A]	N=266; mean=1459.9; SD=10.0; median=1461; 50% RI=1458–1463; 90% RI=1443–1477	B2011-T4; PDF p. 32 (article p. 043101-32)	Dimethylsilicone stationary phase with 5% phenyl groups; published statistical compilation	1460	Published mean 1459.9 rounded to 1460 (nearest integer; 0.5 upward).
51	γ-Muurolene [ID A]	N=292; mean=1476.2; SD=8.9; median=1477; 50% RI=1474–1479; 90% RI=1461–1487	B2011-T4; PDF p. 32 (article p. 043101-32)	Dimethylsilicone stationary phase with 5% phenyl groups; published statistical compilation	1476	Published mean 1476.2 rounded to 1476 (nearest integer; 0.5 upward).
52	Unidentified AO-16 (best MS match: Germacrene B; retention conflict) [ID C]	B2011 Germacrene B: N=177; mean=1550.9; SD=18.9; median=1556; 50% RI=1552–1560; 90% RI=1499–1568. Present NIST hit 1: SI=91.	B2011-T4, PDF p. 33; original AO PDF, peak 16.	B2011 5%-phenyl statistical record; present peak assessed by Rxi-5Sil MS elution order.	n.a.	Germacrene B should not elute before β-bisabolene (RI 1508) if its expected RI is about 1551. The peak is retained only as an unidentified candidate (ID C); no candidate RI is shown in Table 1.
53	α-Amorphene [ID B]	N=45; mean=1482.4; SD=12.1; median=1483; 50% RI=1474–1485; 90% RI=1466–1506	B2011-T4; PDF p. 32 (article p. 043101-32)	Dimethylsilicone stationary phase with 5% phenyl groups; published statistical compilation	1482	Published mean 1482.4 rounded to 1482 (nearest integer; 0.5 upward). The compound assignment remains tentative (ID B).
54	β-Bisabolene [ID A]	N=243; mean=1508.4; SD=9.0; median=1509; 50% RI=1506–1510; 90% RI=1494–1525	B2011-T4; PDF p. 33 (article p. 043101-33)	Dimethylsilicone stationary phase with 5% phenyl groups; published statistical compilation	1508	Published mean 1508.4 rounded to 1508 (nearest integer; 0.5 upward).
55	Unidentified CO-14 (best MS match: β-copaene; retention conflict) [ID C]	B2011 β-copaene: N=53; mean=1433.1; SD=7.3; median=1432; 50% RI=1430–1433; 90% RI=1426–1449. Present NIST hit 1: SI=91.	B2011-T4, PDF p. 32; original CO PDF, peak 14.	B2011 5%-phenyl statistical record; present peak assessed by Rxi-5Sil MS elution order.	n.a.	The peak elutes between β-bisabolene (RI 1508) and δ-cadinene (RI 1523), which conflicts with β-copaene near 1433. It is therefore unidentified (ID C); lower-ranked candidates are insufficient for reassignment.
56	δ-Cadinene [ID A]	N=551; mean=1523.2; SD=10.1; median=1524; 50% RI=1520–1525; 90% RI=1508–1539	B2011-T4; PDF p. 33 (article p. 043101-33)	Dimethylsilicone stationary phase with 5% phenyl groups; published statistical compilation	1523	Published mean 1523.2 rounded to 1523 (nearest integer; 0.5 upward).

Row link	Table 1 entry	Literature/database source evidence (unaltered)			Reporting information for the revised Table 1	
**Table 1 No.**	**Compound / ID**	**Original reported value(s)**	**Source ID and exact location**	**Stationary phase / RI record**	**RI reported in Table 1**	**Reporting basis / identification assessment**
57	Unidentified CO-16 (best MS match: 4,8,11,11-tetramethyltricyclo[7.2.0.0(3,8)]undec-4-ene) [ID C]	— (no source value adopted)	—	—	n.a.	No literature/database RI was adopted because the peak remains unidentified (ID C); a library candidate or RI_NIST in S1 is traceability evidence only, not an assigned compound RI.
58	2-Methoxycinnamaldehyde (tentative) [ID B]	NIST-SRD69/Marongiu et al. 2007: RI=1505.	NIST-SRD69 C1504741; MAR2007.	DB-5MS, 30 m × 0.25 mm × 0.25 µm, He; 60 °C, 3 °C/min to 280 °C; normal-alkane temperature-ramp RI.	1505	The directly traceable DB-5MS value was adopted. Because the present elution position is slightly later than expected and no same-run RI was measured, the assignment is retained as ID B.
59	(E)-Nerolidol [ID A]	N=232; mean=1560.9; SD=9.1; median=1564; 50% RI=1560–1565; 90% RI=1539–1570	B2011-T4; PDF p. 33 (article p. 043101-33)	Dimethylsilicone stationary phase with 5% phenyl groups; published statistical compilation	1561	Published mean 1560.9 rounded to 1561 (nearest integer; 0.5 upward).
60	Caryophyllene oxide [ID A]	N=498; mean=1580.6; SD=9.8; median=1581; 50% RI=1578–1583; 90% RI=1563–1595	B2011-T4; PDF p. 33 (article p. 043101-33)	Dimethylsilicone stationary phase with 5% phenyl groups; published statistical compilation	1581	Published mean 1580.6 rounded to 1581 (nearest integer; 0.5 upward).
61	α-Cadinol [ID B]	N=297; mean=1651.9; SD=9.5; median=1653; 50% RI=1651–1655; 90% RI=1635–1664	B2011-T4; PDF p. 34 (article p. 043101-34)	Dimethylsilicone stationary phase with 5% phenyl groups; published statistical compilation	1652	Published mean 1651.9 rounded to 1652 (nearest integer; 0.5 upward). The compound assignment remains tentative (ID B).
62	Foeniculin [ID B]	BOS2024 foeniculin: RI_exp=1676; RI_lit=1676. MUR2024 foeniculine: KI=1677.	BOS2024-T2, PDF p. 7 (printed p. 133); MUR2024-T1/T2, PDF pp. 3–4.	BOS2024: 30 m DB-5 with C8–C40 n-alkanes for RI_exp. MUR2024: Adams linear DB-5 KI.	1676	The directly measured DB-5 RI_exp=1676 was adopted; the independent KI=1677 is reported separately and was not averaged. Assignment remains tentative (ID B).
63	Unidentified CO-20 (best MS match: β-selinene; retention conflict) [ID C]	N=256; mean=1486.1; SD=8.7; median=1486; 50% RI=1484–1490; 90% RI=1473–1496	B2011-T4; PDF p. 32 (article p. 043101-32)	Dimethylsilicone stationary phase with 5% phenyl groups; published statistical compilation	n.a.	A reference value exists for the library candidate, but the supplied peak has a marked retention/elution conflict and remains ID C. The former 1486 dagger entry was removed from Table 1.
64	trans-Geranylgeraniol [ID B]	NIST-SRD69/Tret'yakov 2007: I=2201.2. EHL2021 Table 3: (E,E,E)-geranylgeraniol RI=2190.	NIST-SRD69 C24034739; TRET2007; EHL2021, Table 3.	VF-5MS, 30 m × 0.25 mm × 0.25 µm, He; 60–270 °C multistep program; Van den Dool and Kratz RI. EHL2021 provides an independent peer-reviewed RI value.	2201	The method-matched VF-5MS value 2201.2 was selected and rounded to 2201 for the RI column in Table 1; the independent published value 2190 is shown without averaging. The library similarity is only 88 and no same-run RI is available, so the assignment remains ID B.

Row link	Table 1 entry	Literature/database source evidence (unaltered)			Reporting information for the revised Table 1	
Table 1 No.	Compound / ID	Original reported value(s)	Source ID and exact location	Stationary phase / RI record	RI reported in Table 1	Reporting basis / identification assessment
65	Unidentified LCO-28 (best MS match: isoaromadendrene epoxide) [ID C]	— (no source value adopted)	—	—	n.a.	No literature/database RI was adopted because the peak remains unidentified (ID C); a library candidate or RI_NIST in S1 is traceability evidence only, not an assigned compound RI.
66	Unidentified LCO-29 (best MS match: neric acid) [ID C]	— (no source value adopted)	—	—	n.a.	No literature/database RI was adopted because the peak remains unidentified (ID C); a library candidate or RI_NIST in S1 is traceability evidence only, not an assigned compound RI.
67	Unidentified LCO-30 [ID C]	— (no source value adopted)	—	—	n.a.	No literature/database RI was adopted because the peak remains unidentified (ID C); a library candidate or RI_NIST in S1 is traceability evidence only, not an assigned compound RI.

Note:

The blue block contains source evidence; the yellow block contains the reporting information selected for the revised Table 1. The two blocks must not be read interchangeably. Values in the source-evidence block are transcribed from the cited publication/database record and were not recalculated.

For B2011, mean, SD, median, 50% RI range, and 90% RI range are shown only when printed in Table 4; 'not reported' indicates a blank field in the source table. The RI value reported in Table 1 is rounded from the published mean, where stated, and the rounding decision appears only in the reporting-information block.

For ID C rows, candidate RI values are retained only in the source evidence to explain exclusion; the RI reported in Table 1 is n.a. and the candidate value does not constitute identification.

RI_exp values appearing in S2 belong to the cited external publications. They are not experimental RI values for the four chromatograms analyzed in the present study.

Source records cited in Supplementary Table S2

B2011. Babushok VI, Linstrom PJ, Zenkevich IG. Retention Indices for Frequently Reported Compounds of Plant Essential Oils. J Phys Chem Ref Data. 2011;40:043101. doi:10.1063/1.3653552. Table 4, PDF pp. 27–34.

RESTEK. Rxi-5Sil MS GC Capillary Column, catalog no. 13623, 30 m × 0.25 mm ID × 0.25 µm; Restek technical description of the silarylene 5-type stationary phase. https://www.restek.com/p/13623. Accessed 25 July 2026.

ORO2020. Oro V, Krnjajic S, Tabakovic M, Stanojevic JS, Ilic-Stojanovic S. Nematicidal Activity of Essential Oils on a Psychrophilic Panagrolaimus sp. Plants. 2020;9:1588. doi:10.3390/plants9111588.

LIU2013. Liu XC, Li YP, Li HQ, Deng ZW, Zhou L, Liu ZL, Du SS. Identification of Repellent and Insecticidal Constituents of the Essential Oil of Artemisia rupestris L. Aerial Parts against Liposcelis bostrychophila Badonnel. Molecules. 2013;18:10733–10746. doi:10.3390/molecules180910733.

NIST-SRD69. NIST Chemistry WebBook, Standard Reference Database 69, gas-chromatographic retention-index records, including C91645 (coumarin), C1504741 (2-methoxycinnamaldehyde), and C24034739 (trans-geranylgeraniol). doi:10.18434/T4D303. Accessed 25 July 2026.

MAR2007. Marongiu B, Piras A, Porcedda S, Tuveri E, Sanjust E, Meli M, Sollai F, Zucca P, Rescigno A. Supercritical CO2 extract of Cinnamomum zeylanicum: Chemical characterization and antityrosinase activity. J Agric Food Chem. 2007;55:10022–10027. doi:10.1021/jf071938f.

BOS2024. Bošković D, Baser N, Vuković S, et al. Evaluation of Pimpinella anisum essential oils on egg-laying behaviour of Drosophila suzukii: A multiple-choice test approach. Agro-knowledge Journal. 2024;25:127–138. doi:10.7251/AGREN2402127B.

MUR2024. Murphy BJ, Wilson TM, Ziebarth EA, Bowerbank CR, Carlson RE. Authentication of Fennel, Star Anise, and Anise Essential Oils by Gas Chromatography (GC/MS) and Stable Isotope Ratio (GC/IRMS) Analyses. Plants. 2024;13:214. doi:10.3390/plants13020214.

Adams RP. Identification of Essential Oil Components by Gas Chromatography/Mass Spectrometry. 4th ed. Carol Stream, IL: Allured Publishing; 2007.

van den Dool H, Kratz PD. A generalization of the retention index system including linear temperature programmed gas-liquid partition chromatography. J Chromatogr. 1963;11:463–471. doi:10.1016/S0021-9673(01)80947-X.

TRET2007. Tret'yakov KV. Retention Data. NIST Mass Spectrometry Data Center; 2007. Cited in NIST-SRD69 record C24034739.

EHL2021. Ehlers S, Szczerbowski D, Harig T, Stell M, Hötling S, Darragh K, Jiggins CD, Schulz S. Identification and Composition of Clasper Scent Gland Components of the Butterfly Heliconius erato and Its Relation to Mimicry. ChemBioChem. 2021;22:3300–3313. doi:10.1002/cbic.202100372.
